# Conditional effects of local and global risk factors on the co-movements between economic growth and inflation: Insights into G8 economies

**DOI:** 10.1016/j.heliyon.2023.e19570

**Published:** 2023-08-31

**Authors:** Emmanuel Asafo-Adjei, Thobekile Qabhobho, Anokye M. Adam

**Affiliations:** a Department of Economics, Faculty of Business and Economic Sciences, Nelson Mandela University, Port Elizabeth, South Africa; b Department of Finance, School of Business, University of Cape Coast, Cape Coast Ghana

**Keywords:** Geopolitical risk, Economic policy uncertainty, Economic events, Connectedness, Bi-wavelet, Partial wavelet

## Abstract

World economies have experienced rise in uncertainties which has caused misalignments in the already existing nexus between inflation and economic growth. In addition to this, the presence of nonlinearities, asymmetry, heterogeneity, and structural shocks in time series data concerning substantial fluctuations that span systemic crises have rendered time and/or frequency connectedness worthy of investigation. Due to limited studies in this regard, the authors investigated the risk synchronisation among Gross Domestic Product (GDP), Consumer Price Index (CPI), Economic Policy Uncertainty (EPU) and Geopolitical Risk with insights from G8 countries. To achieve the study's purpose, estimation techniques employed included the wavelet approaches (bi-wavelet and partial wavelet), and the wavelet multiple as well as the DCC-GARCH Connectedness approach as robustness. A sample period from January 1997 to August 2021 restricted by consistent data availability was considered. It was discovered that most G8 nations have a comparable relationship between their GDP and CPI. Additionally, significant co-movements between the G8 nations' GDP and CPI straddle crises. Furthermore, the relationship between Russia's GDP and CPI was significantly conditionally influenced by geopolitical risk factors. Own country economic policy uncertainty was the main source of shocks for nations like Canada, France, and the US, whereas, in Germany, Italy, and the UK, Global EPU was a crucial conduit for reducing the lead-lag relationship between GDP and CPI. Outcomes from this study imply that uncertainties pose a more persistent and dynamic challenge to the G8 countries' efforts to achieve sustained economic growth, lessen the negative effects of inflation and deflation, and improve national and regional economic integration.

## 1 Introduction

For policymakers, achieving both high and sustained economic growth and low inflation becomes a top priority. This is true since the primary factor influencing the economy's rate of expansion is price stability [1]. Not just policymakers are concerned about this association; individuals and companies are equally concerned. At the individual level, inflation has an impact on people with fixed incomes and, in comparison, favours borrowers at the expense of creditors. Additionally, when businesses must maintain costs as they adjust to the new price level, or change their price lists for customers, the average change in price influences output at the firm level [2]. By making a country's exports relatively more expensive, inflation reduces the state's ability to compete internationally. This has an impact on the current account. Furthermore, the levy system and inflation work together to distort borrowing and lending decisions [3]. Political intervention and divergent policy stances continue to make it difficult to establish and maintain a steady inflation rate. The deception that inflation causes in the financial market, labour market, investment, saving, and global competitiveness negatively impacts economic wellbeing as well as the long-term growth rate of the economy [4].

To this end, the nature of the relationship between inflation and economic growth has been extensively investigated and is a contentious topic because of these policy consequences. However, the findings remain contradictory. Sidrauski [5] concluded that there is no correlation between inflation and economic growth; Fischer [6] found a negative correlation, whereas Mallik and Chowdhury [7] found a positive correlation. Although there is still much debate, a substantial number of studies have concluded that inflation has a detrimental medium- and long-term effect on economic growth [6,8,9]. The existence and nature of this interaction, in any case, is a key question, as the large body of extant literature attests. Recent studies thread support to the existence of nonlinearities, implying the rate of inflation determines economic growth and can either improve or worsen depending on whether the threshold is exceeded or not [[10], [11], [12], [13]].

Even though the negative effects of inflation on growth in industrial and developed countries, especially, in the medium-to long-term have been validated beyond reasonable doubt by the majority of previous studies [8], the subject of the effects of the external shock on the co-movements between inflation and economic growth is not addressed in the literature. Hence, the current study purposes to investigate this phenomenon in G8 economies (Canada, Italy, France, United Kingdom, Germany, Japan, Russia, and the US). Global economic policy uncertainty (GEPU) and Geopolitical risk indices are used to proxy external uncertainty shock variables. It is important to note why this study should pay particular attention to G8 nations. Of course, it should be remembered that these nations are among the most powerful in terms of their economy and politics and that any instability in their economies has an influence on other economies around the globe. As a result, policymakers in other nations base their judgments on the economic patterns of powerful economies like the G8 groupings.

The fact that countries are experiencing a high level of uncertainty as a result of global tensions, terrorist attacks, geopolitical risk, and global economic uncertainty makes the high levels of inflation and sluggish economic growth even worse. The persistent consequences of the pandemic and Russia's invasion of Ukraine are harming the global economy. Because of higher-than-expected inflation, particularly in the United States and major European countries, financial conditions around the world are becoming increasingly tight. China's recession has been worse than anticipated because to COVID-19 breakouts and lockdowns, and the situation in Ukraine has had further negative impacts [14]. As a result, the world's output decreased in the second quarter of 2022 [15].

Historically, times of increased geopolitical risk (GPR) have been linked to detrimental consequences on world economic activity. On the supply side, conflicts obliterate human and material resources, redirect resources to less productive purposes, disrupt international trade and capital flows, and disrupt supply networks on a global scale. On the demand side, a lack of understanding regarding the potential consequences of unfavourable geopolitical developments could hinder activity by preventing businesses from investing in new projects and recruiting staff, undermining consumer confidence, and tightening financial conditions. While demand and supply pressures are normally harmful to economic activity, their combined effect on inflation is less clear-cut since the deflationary influences of declining aggregate demand may outweigh the inflationary implications on the supply side.

It is also evident that short-term economic swings are common after a crisis in many different countries. This short-term instability limits the economy's potential to grow further in several ways. A stable macroeconomic climate is necessary for high and sustainable economic growth. To realise sustainable economic growth, we must carefully examine the causes of short-term economic instability. Uncertainty in economic policy has a significant negative effect on the expansion of a sizable economy, among other things. This is mostly demonstrated by the fact that governments frequently adopt policies to support economic recovery, oblivious to the fact that doing so may exacerbate macroeconomic oscillations or even have the opposite impact of what was intended.

We hope to contribute to both the policy and scientific fields by looking at this connectedness. It is essential for policymakers and economists to comprehend the complex dynamics between economic growth, inflation, and external factors to develop successful plans and programs. Geopolitical risk and economic policy uncertainty have drawn attention recently for their potential to have an impact on economic results. By identifying the precise ways in which economic policy uncertainty and geopolitical risk affect the connectedness between economic growth and inflation, this research aims to offer useful insights to policymakers. Such information can help policymakers in the G8 economies reduce risks, promote stability, and advance sustainable economic growth. Scientifically, this study adds to the existing literature by advancing our knowledge of the complex interdependencies between country-specific macroeconomic variables and external shocks across time and/or frequency. The findings will enhance the understanding of economic dynamics in G8 economies and provide a foundation for further research in this field.

This study provides three distinct contributions regarding the objective of investigating the conditional impact of geopolitical risk and economic policy uncertainty on the co-movements between economic growth and inflation in G8 economies. First, using a bi-wavelet, we examine how the link between inflation and economic growth in G8 economies changes across time and frequency. The existing literature that studies the link between inflation and economic growth pays less attention to time-frequency analysis, which could have an impact on the findings when assessing this phenomenon. Second, the partial wavelet displays the co-movements between GDP and CPI relative to a common interdependence captured in this study as external uncertainty shocks. By doing so, we will be able to reveal the conditional impact of EPU and GPR on the nexus between GDP and CPI. Moreover, we assess the connectivity among the variables for important net-transmitters and net-receivers using the two-step DCC-GARCH approach which is devoid of a rolling window framework [16] relative to the Diebold and Yilmax [17].

To present a complete picture of the nexus, the wavelet multiple approach is also utilised as a robustness check to identify the leading/lagging variable relative to the scales through linear combinations for more than two variables across intrinsic time horizons. For several reasons, we focus on the link between inflation which we represent by average change in prices/or Consumer Price Index (CPI) and economic growth (which we proxy as Gross Domestic Product) over time while controlling for external uncertainty shocks. Hence, as previously indicated, we define external uncertainty shocks to mean GPR and GEPU.

It was revealed that the majority of G8 countries' GDP and CPI have similar relationships. Significant co-movements between the GDP and CPI of the G8 countries also cross crises. Geopolitical risk variables also have a major conditional impact on the link between Russia's GDP and CPI. For countries like Canada, France, and the US, uncertainty in domestic economic policy was the main cause of shocks, whereas in Germany, Italy, and the UK, GEPU was a key channel for lowering the lead-lag relationship between GDP and CPI. Results from the DCC-GARCH Connectedness approach showed that, on average, GDP and CPI of G8 economies are net receivers of shocks in the network of selected macroeconomic variables, with the exception of CPI of Russia.

The remaining sections are presented focusing on areas such as the Literature Review, Methodology, Results and Discussion and Conclusions.

## 2 Literature review

Researchers and policymakers have recently focused on concerns about Economic Policy Uncertainty (EPU), the effects of different economies on one another, the global financial crisis, and geopolitical upheavals since the economies of many nations seek deeper linkages with the global economy [[18], [19], [20]]. Economic Policy Uncertainty (EPU) is the term for instability brought on by shifts in governmental economic policies. Important decisions concerning employment, investment, consumption, corporate savings, and other economic matters may be delayed or altered as a result. Therefore, the consequences of economic instability are borne by the economies [21]. The Economic Policy Uncertainty (EPU) index can be measured in several different ways [22]. The first method is to study stock market volatility, which establishes how much volatility will be in a certain period in the pertinent horizons.

Another method is based on newspaper articles and various public and economic reports reviews. The number of words strongly related to uncertainty that is used in newspapers, economic reports, and other public publications can be counted and examined to determine the Economic Policy Uncertainty (EPU) index. In addition, to generate this index, any tweets that contain the words "economics" and "uncertainty" are considered [23]. The goal of acquiring and studying this index is to put future economic policy implementation and investor economic decisions into perspective. The Global Economic Policy Uncertainty (GEPU) index, which is the global EPU index, is created by weighting the Economic Policy Uncertainty (EPU) index of the sixteen developed nations throughout the world. About 70% of the global GDP is made up of these sixteen nations' GDPs. It demonstrates the degree of economic apprehension and investor faith in economic indicators for potential investment [24].

Geopolitical risk is another concerning factor that is driving up research in economics and finance literature. Flint [25] defines geopolitical risk as any type of power conflict and includes dangers related to armed warfare, terrorist acts, and conflicts between and within governments that cannot be resolved by peaceful democratic means. Geopolitical risk, according to Zhang and Hamori [26], occurs when more military or political conflicts in a particular region damage the local economy or even the global economy. For instance, terrorism or war may lead to rising costs, dwindling wages, or a sluggish global economy. An example would be the rise in energy prices brought on by Russia's invasion of Ukraine, which eventually causes inflation. Geopolitical risk, according to Balcilar et al. [27] has the potential to alter business cycles, impede investments, and, as a result, have an impact on the economy of the nation in question. Comparing the EPU and GPR indices, Caldara and Iacoviello [28] contend that the geopolitical risk index captures events that are more exogenous to business and financial cycles, which could exacerbate economic instability.

Empirically, this line of inquiry has been the subject of numerous studies that aim to provide answers to the question of what impact external factors such as global economic policy uncertainty and geopolitical risks (terrorist attacks, wars, revolutions, and political instability) have on inflation and economic growth. Alesina and Perotti [29], for instance, demonstrate how political unrest inhibits economic growth in 72 different nations over the period 1960–1985 using a regression model. They contend that a fall in investment because of political upheaval leads to a slowdown in economic growth and a worsening of income disparity. Using monthly data for the period of 1974–2022, Caldara, Conlick, Iacoviello, and Penn [30] investigated whether geopolitical risk increased or decreased inflation. They discovered that elevated geopolitical risk could lead to an uncertain outlook for inflation and higher inflation upside risks. They also showed how the trade-offs that fiscal and monetary policy must make as a result of growing inflation and a decline in economic activity have been made worse by the Russian invasion of Ukraine. In the same vein, Soybilgen et al. [31] examine the close relationship between geopolitical threats and the relevant economic growth variable for a panel of 18 emerging nations over the period 1986–2016. They made use of the Caldara and Iacoviello [28] GPR index. Their findings demonstrate that geopolitical risk is negatively impacting economic and human growth.

Barros Jr, Gomes, and Soave [32] looked into the effects of global geopolitical risk shocks on the Brazilian economy using quarterly data from Brazil as well as indices that take into account GPR connected to the US, Russia, and a worldwide GPR index. In terms of their findings of a Bayesian vector autoregressive model, real economic activity reacts more strongly to Russian risk than to global or US risk, despite the fact that prices and financial indicators seem to be more responsive to global risk. Baker, Bloom, and Davis [23] created country-specific indexes of uncertain economic policy for United States of America and 12 major economies and looked at how the indexes affected the economies of those countries. It has been shown that the US index peaks after crucial political events including presidential contests, wars, the collapse of Lehman Brothers, and various disputes over fiscal policy. Using macro-level data, their findings further indicate that increased policy uncertainty in the United States and Europe has impacted macroeconomic performance. Their results also point to significant implications of policy uncertainty on the cross-sectional pattern of stock market volatility, investment rates, and employment growth. Using the linear and nonlinear autoregressive distributive lag (ARDL) approach, Wen, Khalid, Mahmood, and Yang [33] also looked at the effects of economic policy uncertainty (EPU) on economic growth for the period 2011M1-2020M5 in Pakistan. Their findings from the nonlinear (NARDL) model demonstrate that high EPU shocks have a detrimental effect on short-run economic growth. They also contend that countries with issues like non-diverse sectors, price spikes, unstable politics, and weak economic and financial structures are particularly susceptible to this negative effect.

Furthermore, using a BVAR model, Istrefi and Piloiu [34] evaluate the effects of economic policy uncertainty on inflation expectations in the US and eurozone countries from 1999Q1 to 2012Q3. The findings demonstrate that policy-related uncertainty shocks have an impact on both long-term and short-term inflation expectations. Nyawo and van Wyk [35] used a structural VAR model to analyse the effects of a shock to US economic policy uncertainty on Indian macroeconomic indicators. Their findings demonstrate that the US economic policy uncertainties cause shocks that statistically significantly lower Indian industrial production and inflation.

The literature reviewed show that geopolitical risk and economic policy uncertainty (both country-specific and global economic policy uncertainty indices) have a significant direct impact on economic growth and inflation. In contrast to the studies previously mentioned, the current study is intended to look at how domestic (country-specific EPU and GPR) and global risk factors (GEPU and GPR) may affect the co-movements of inflation which we represent by Consumer Price Index (CPI), and economic growth captured by Gross Domestic Product (GDP) under certain conditions. We employ the bi-wavelet for assessing co-movements, partial wavelet for conditional impact analysis, and wavelet multiple correlations for methodically and meticulously determining the strength and direction of the connections between GEPU, GPR, CPI, and GDP across scales. Additionally, in comparison to the Diebold and Yilmaz [17] technique, we evaluate the connectedness between the variables among significant net-transmitters and net-receivers utilizing the two-step DCC-GARCH based TVP-VAR approach that is free of a rolling window framework. Hence, the following research questions are found.

1. What is the co-movement between economic growth and inflation rate of G8 economies across time and frequency?
2. What is the partial influence of uncertainties on the co-movements between economic growth and inflation rate of G8 economies across time and frequency?
3. What is the degree of dynamic integration among economic growth, inflation and uncertainties?
4. Does local induced uncertainty matter most for G8 macroeconomic fundamentals than global risk factors?

## 3 Methodology

### 3.1 Data sources and description

The study employed monthly time series data on gross domestic product (GDP) constructed by the Organisation for Economic Corporation and Development (OECD) to gauge the short-term dynamics developed from chained volume estimates of quarterly GDP series in US dollars and Consumer Price Index (CPI) for G8 countries. The countries included Canada (CD), Italy (ITA), France (FRA), United Kingdom (UK), Germany (GER), Japan (JPN), Russia (RUS) and the United States (US). Additionally, we used external shock factors such as Global economic policy uncertainty (GEPU) and Geopolitical risk (GPR) to divulge their conditionality on the nexus in the macroeconomic fundamentals of the G8 economies. The choice of the variables was motivated from several sources, for instance, GDP and CPI [7,[9], [10], [11], [12], [13],^36^], GPR [14,[25], [26], [27], [28],^31^,^32^], and EPU [18,22,24,34]. In line with several economic events world economies have witnessed so far, we allowed a sample period from January 1997 to August 2021 restricted by consistent data available for the selected variables. The final data on the chosen variables were merged to have common dates for a direct comparative analysis across time and/or frequency using codes in R programming. The data on GDP and CPI were sourced from OECD database whereas on GEPU and Geopolitical risk were gleaned from the website https://www.policyuncertainty.com/index.html. Because the chosen techniques can deal with nonstationary, nonnormal and nonlinear problems, analyses are performed on the raw series without transformation.

### 3.2 Wavelet methods

#### 3.2.1 Bi-wavelet

In this study, the bi-wavelet technique is utilised to investigate the lead-lag relationship between the GDP and CPI of G8 economies from a time and frequency perspective. The time and frequency in this case include calendar times and intrinsic times of short-, medium-, and long-terms. The bi-wavelet approach in the context of the study has a benefit of addressing significant co-movements between GDP and CPI. The significant co-movements that occur at systemic crises can be considered as contagion effects whereas significant co-movements aside systemic crises indicate the presence of interdependencies.

Torrence and Compo [37] define wavelet transformation coherence (WTC) as the squared value normalization of a cross-absolute spectrum to a single wavelet power spectrum. Equation (1) is the equation for the squared wavelet coefficient;

(1) $$R_{p}^{2}\left(x,y\right)=\frac{{\left|\rho \left(s^{-1}W_{xy}\left(\Omega ,s\right)\right)\right|}^{2}}{\rho \left(s^{-1}{\left|W_{x}\left(\Omega ,s\right)\right|}^{2}\right)\rho \left(s^{-1}{\left|W_{y}\left(\Omega ,s\right)\right|}^{2}\right)}$$

where ρ is a smoothing factor and the square difference ranges from 0 to 1. A number near to 1 indicates a strong connection, whereas a number close to 0 indicates a weak connection. The statistical significance of this nexus was tested using the Monte Carlo method because it is challenging to determine the theoretical distribution of coefficients [37].

The wavelet coherence map's phase pattern dimension draws attention to the wavelet coherence difference as a source of inspiration. To distinguish phase patterns, dimensional arrows are utilised.

Arrows pointing right and left, upward and downward, as well as up and down, are used to graphically represent the bi-wavelet. Right arrows pointing up and left arrows pointing down indicate the first variable, and vice versa for left arrows pointing up and right arrows pointing down. The relationship between the linked variables is demonstrated using a color scheme and a surface colour. Areas with a lot of co-movements are shown in red (warm), whereas those with less co-movements are shown in blue (cool) [38]. Outside of the sphere of influence, the results are unimportant (COI).

#### 3.2.2 Partial wavelet coherence (PWC)

The PWC is employed in the literature to limit the problem of "pure" correlation between time-series variables z(t) and to control the influence of a time series variable on the wavelet coherence between other two time series variables x(t) and y(t). In the context of the study, the conditional effects of external shocks (GPR and GEPU) on the nexus amid GDP and CPI of G8 nations are investigated. As illustrated in equation (2), PWC is represented by a similar equation to partial correlation squared;

(2) $$R_{p}^{2}\left(x,y,z\right)=\frac{{\left|R\left(x,y\right)-R\left(x,z\right)•R{\left(x,y\right)}^{•}\right|}^{2}}{{\left[1-R\left(x,z\right)\right]}^{2}\;{\left[1-R\left(y,z\right)\right]}^{2}}$$

where $R_{p}^{2}\left(x,y,z\right)$ is between 0 and 1. In the study, the letters x, y, and z stand for the GDP, the CPI, and external shock factors (GPR and GEPU), respectively. For estimating, PWC employs Monte Carlo techniques.

## 4 Results and discussion

### 4.1 Preliminary statistics

**Fig. 1:**
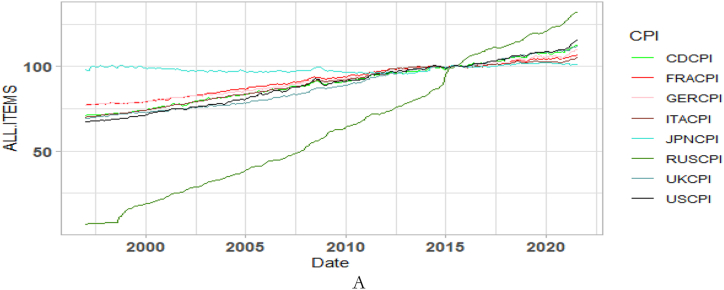

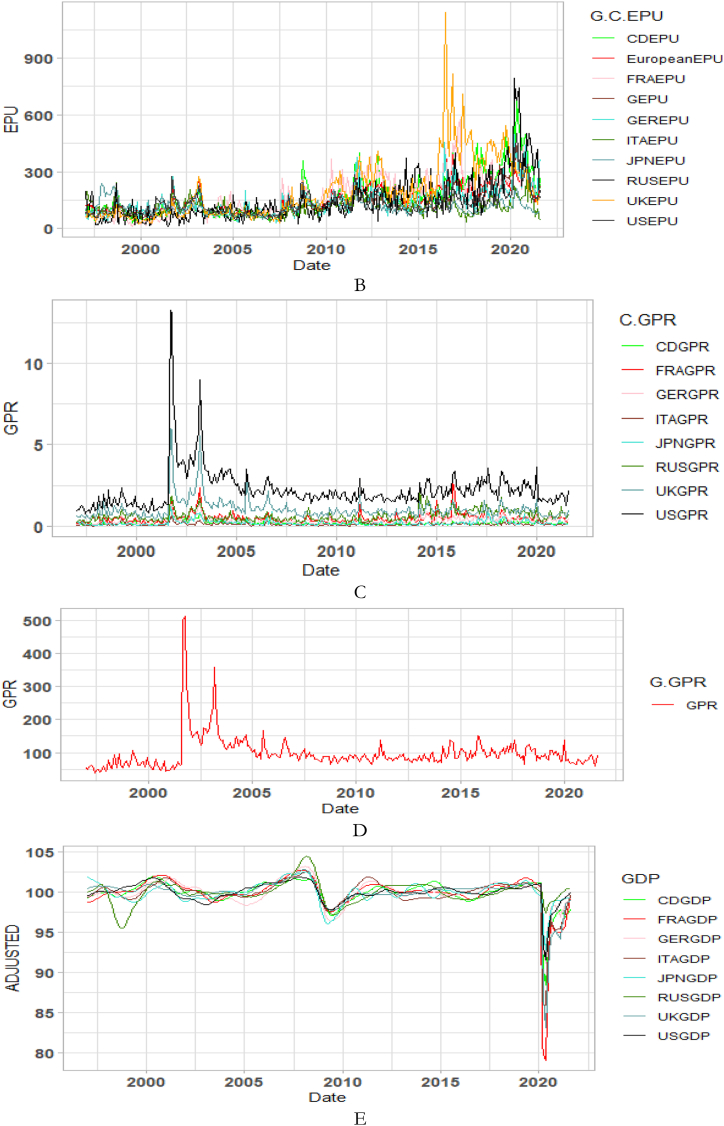
Time series plots. Panel A: CPI of G8 countries; Panel B: Global and country-specific economic policy uncertainty; Panel C: GPR measure of G8 countries; Panel D: Global GPR; Panel E: GDP of G20 nations. Note: The acronyms G.C.EPU, C.GPR, and G.GPR represent Global and country-specific EPU, country-specific GPR, and Global GPR.

Fig. 1 presents a trajectory of country-specific macroeconomic indicators, global GPR and GEPU spanning 1997 to 2021. The period marks known economic events and shocks such as the 2008 Global Financial crisis (GFC), Arab Spring 2013 Eurozone debt crisis (EZC), BREXIT in 2016, and COVID-19 Pandemic, to name a few, which have majorly altered the long-term movements in the series over the years. The CPI for each country in Fig. 1(A) exhibits an upward trend with serious implications for economic growth [39,36]. The CPI depicts an upsurge in the average change in prices from 1997 to 2021. The similar trends in the time series data among the G8 economies for CPI in Fig. 1(A), EPU in Fig. 1(B), GPR in Fig. 1(C) and GDP in Fig. 1(E) further depict comparable macroeconomic fundamentals within the G8 economies [40]. The countries’ GPR, especially, that of the US, and the global GPR in Fig. 1(D) can be observed to move in similar patterns to suggest that the global index captures most of the dynamics of significant wars, terrorist acts, and tensions-related risks emanating from the G8 economies.

We focus on the preliminary statistics from Table 1 for the G8 countries as well as global and continental shocks. It can be observed from Table 1 that the average value of CPI for Japan is the highest, followed by France, and closely by Germany. As presented, the series are not normally distributed (illustrated by the Jarque-Bera statistic). However, the estimation techniques utilised in this study have a scope of deciphering traits from the series.

**Table 1:** Preliminary statistics among GDP, CPI, EPU and GPR.

Variable	Mean	Std. Dev.	Skewness	Kurtosis	Jarque-Bera
CDCPI	90.406	11.737	−0.035	1.847	16.470^a^
CDEPU	166.731	113.684	1.325	4.825	127.642^a^
CDGDP	99.888	1.617	−3.152	18.750	3549.686^a^
CDGPR	0.204	0.161	5.482	45.740	24012.680^a^
FRACPI	92.320	9.052	−0.225	1.688	23.729^a^
FRAEPU	171.370	104.932	0.697	3.255	24.755^a^
FRAGDP	99.767	2.649	−5.064	36.201	14860.690^a^
FRAGPR	0.505	0.314	3.325	19.073	3731.522^a^
GERCPI	91.966	9.361	0.007	1.713	20.432^a^
GEREPU	142.175	78.217	1.523	5.998	225.247^a^
GERGDP	99.917	1.536	−1.380	8.588	479.177^a^
GERGPR	0.357	0.214	2.272	11.211	1086.000^a^
ITACPI	89.898	10.876	−0.351	1.712	26.536^a^
ITAEPU	112.185	41.436	0.869	3.961	48.666^a^
ITAGDP	99.812	1.995	−3.996	26.035	7332.152^a^
ITAGPR	0.133	0.084	2.215	10.215	884.008^a^
JPNCPI	98.648	1.837	0.293	1.812	21.619^a^
JPNEPU	110.322	36.298	1.171	4.498	95.278^a^
JPNGDP	99.947	1.329	−1.326	7.311	315.985^a^
JPNGPR	0.223	0.162	2.510	11.396	1180.045^a^
RUSCPI	63.506	37.503	0.210	1.777	20.617^a^
RUSEPU	148.333	121.112	2.099	9.241	697.804^a^
RUSGDP	99.915	1.411	−0.006	5.238	61.755^a^
RUSGPR	0.651	0.341	1.498	5.762	204.698^a^
UKCPI	88.732	12.981	0.136	1.600	25.088^a^
UKEPU	193.924	153.568	1.796	8.466	527.543^a^
UKGDP	99.839	2.023	−4.516	30.848	10570.650^a^
UKGPR	1.055	0.658	4.565	30.720	10504.700^a^
USCPI	89.539	13.336	−0.093	1.793	18.383^a^
USEPU	130.762	64.947	2.088	9.647	759.914^a^
USGDP	99.940	1.246	−2.431	14.681	1974.431^a^
USGPR	2.248	1.334	4.726	34.890	13644.640^a^
EUROPEANEPU	152.071	69.179	0.937	4.083	57.749^a^
GEPU	129.984	70.179	1.542	5.345	185.183^a^
GPR	97.968	50.254	4.649	34.060	12964.410^a^

Note: ^a^ depicts significance at 1%.

### 4.2 Co-movements between GDP and CPI of G8 countries

**Fig. 2:**
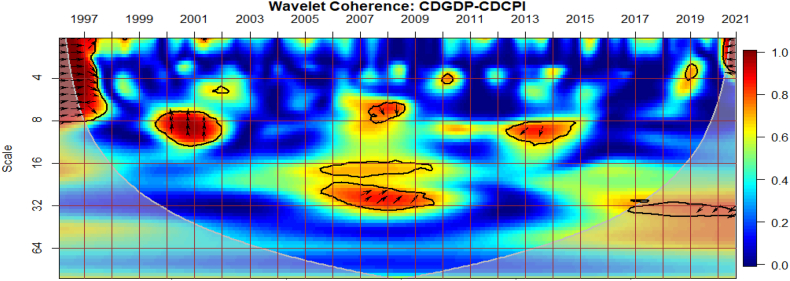

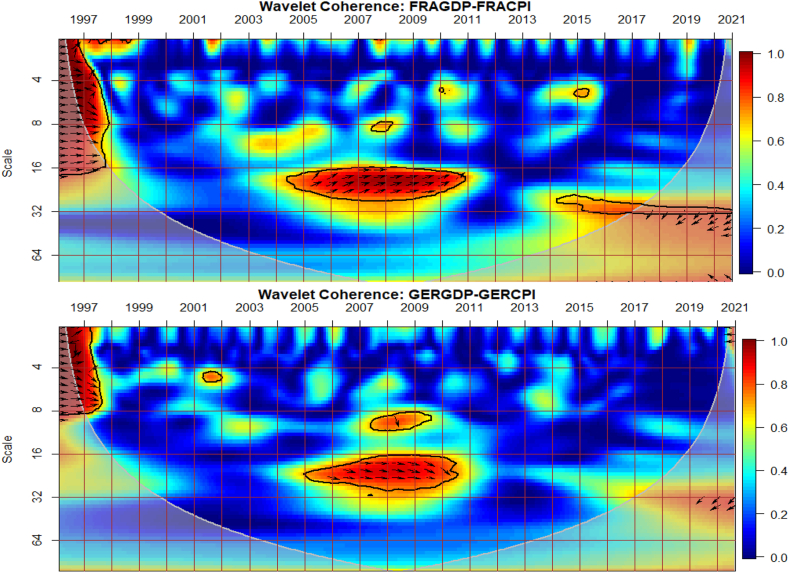

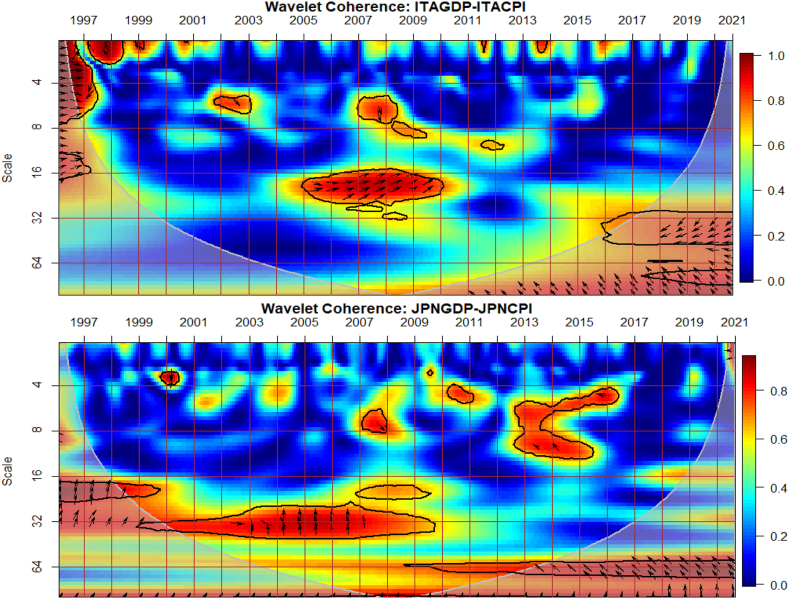

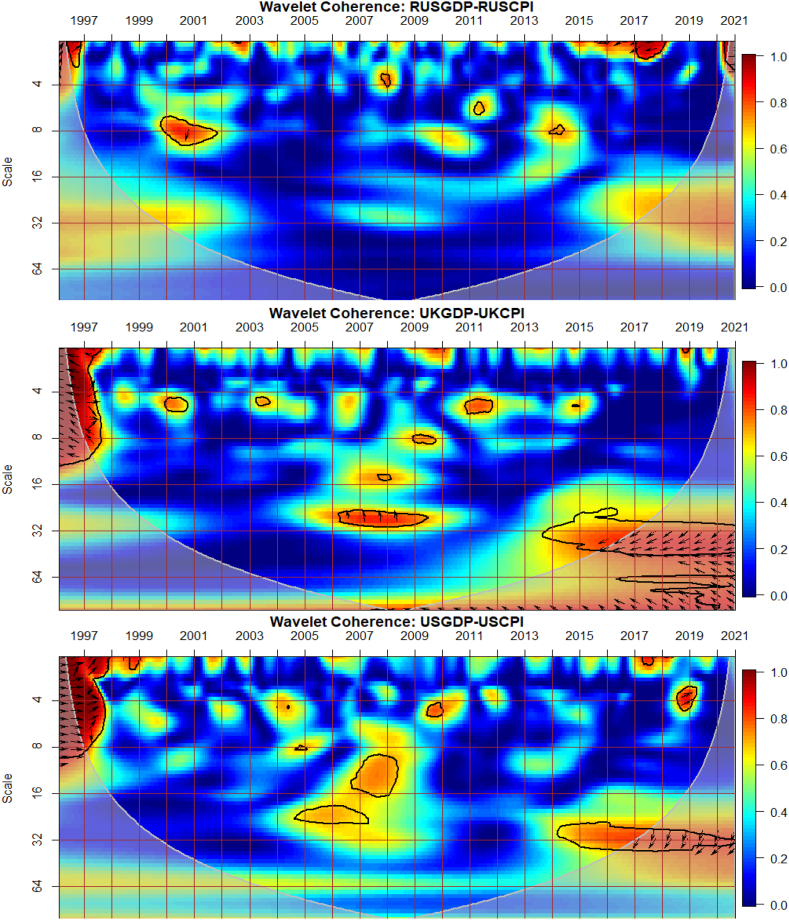
Co-movements between GDP and CPI for each of the G8 countries. Panel A: CDGDP-CDCPI; Panel B: FRAGDP-FRACPI; Panel C: GERGDP-GERCPI; Panel D: ITAGDP-ITACPI; Panel E: JPNGDP-JPNCPI; Panel F: RUSGDP-RUSCPI; Panel G: UKGDP-UKCPI; Panel H: USGDP-USCPI.

The co-movements between GDP and CPI for each of the G8 countries are investigated across time and frequency using the bi-wavelet approach in Fig. 2. We highlight negative and positive nexus, as well as leading and lagging variables across calendar times (to respond to economic events) and intrinsic times (to assess time horizons of short-, medium-, and long-terms) [41] to usher policy implications. As noted from prior studies, right-pointing arrows depict positive nexus suggesting movement in the same direction whereas left-pointing arrows denote negative nexus [41,42]. Substantial or strong connectedness is shown in warm colour but weak connectedness is shown in blue colour. Substantial connectedness between GDP and CPI during economic events or crises suggest contagion effects whereas at normal periods with no known crises, stronger co-movements indicate spillover effects. In this study, GDP was set as the first variable whereas CPI was set as the second variable for all countries to ensure consistency of presentation, although the order of the variables does not matter, and hence, any order can be chosen. The first variable (GDP) leads when the arrows point either to the right-upwards or left-downwards, and otherwise for a lead in the second variable (CPI).

Except for Japan in Fig. 2(E) and possibly, Russia in Fig. 2(F), the remaining G8 countries experienced significant co-movements of GDP and CPI from 1997 to 1998 in the short-term. The significant co-movements during this period are mostly positive as shown by the right-pointing arrows apart from UK in Fig. 2(G). This suggests a similar movement in the direction of both GDP and CPI in most countries. To decipher leading and lagging variables, a twist in the right-pointing arrows either upwards or downwards is examined. For countries like Canada in Fig. 2(A), France in Fig. 2(B), Germany in Fig. 2 (C) and Italy in Fig. 2(D), right-pointing arrows were mostly downwards indicating that the second variable leads the nexus. Accordingly, CPI of these countries was a significant driver and determinant of economic activities. In other words, in the short-term of 1997 and 1998, CPI was a significant predictor of GDP in the said countries. The situation was different in the case of the US in Fig. 2(H), experiencing a drive in CPI by GDP. Co-movements of GDP and CPI in UK was entirely different, recording negative nexus at the initial part of 1997 in the short-term and both positive and negative nexus at the latter part of 1997. The positive and negative co-movements during this period for UK highlights the interdependence structure of the macroeconomic fundamentals which affects economic policy [[42], [43], [44]].

An interesting result is found between 2007 and 2013 representing the 2008 GFC and the 2013 EZC for all G8 countries, except Russia. We find few significant co-movements for US and UK with indeterminable leading or lagging variable. This proposes that in the heat of the known crises, GDP and CPI were less connected in both US and UK whereas the remaining G8 countries, but Russia responded to a higher extent. Particularly for Canada, France and Italy, right-pointing arrows are mostly upwards where GDP led CPI, but vice-versa for Germany. The lead by GDP during the 2008 GFC concurs the outcome of Naghdi, Kaghazian and Kakoei [45] that economic growth was the most affected channel through which financial crisis influenced inflation of G8 countries.

Japan is noticed to exhibit significant co-movements between GDP and CPI in the long-term between 1998 and 2013 with left pointing-arrows upwards. This explains that an increase in CPI drives GDP downwards. Similar dynamics can be observed in the medium-term between 2013 and 2017 for Japan, and can be attributed to the delayed impact [46] of the 2011 Japan's Tsunami and Fukushima Nuclear Disaster [47,48]. The Japanese economy is touted to exhibit chronic deflation and low growth from an attempt to deflate speculation and maintain inflation at appropriate levels by sharply upsurging inter-bank lending rates initiated by the Bank of Japan in late 1989. However, the inverse co-movements found between 1998 and 2017 leads to the transformation of the Japanese economy from a low-inflation/low-growth to either a high-inflation/low-growth or a low-inflation/high-growth economy with CPI as the major determinant.

Conversely to other studies that find significant co-movements between macroeconomic variables during the COVID-19 pandemic [[49], [50], [51], [52]], little is captured by the G8 countries. Nonetheless, we provide support to existing studies that find the heterogeneity and adaptability nature of the GDP-CPI nexus that the rate of inflation determines economic growth and can either improve or worsen it depending on systemic crises era or idiosyncratic risk factors [[10], [11], [12], [13]]. It is also instructive to note that the nexus between GDP and CPI of most G8 countries as highly industrialised are comparable except for Russia which shows less integration with most countries on macroeconomic fundamentals [53]. Nonetheless, there is greater potential for the economies’ capacity to foster harmony on global issues like economic growth, CPI and crisis management.

### 4.3 Susceptibility of the co-movements between GDP and CPI to country level and global shocks

To investigate the impact of shocks from within and outside each G8 economy, the partial wavelet approach is used. The partial impact of economic policy uncertainty and geopolitical risk as a common interdependence in the co-movements between GDP and CPI of the G8 countries is examined. We do this to decipher significant shock transmitters in the co-movements for economic policy implications. For this approach, strong transmitters are denoted by the degree to which already existing co-movements between GDP and CPI are distorted (shown by mild warm or faded warm colour). Specifically, the extent of reduction in the stronger co-movements observed in the bi-wavelet case indicates the substantial impact of the risk factors (geopolitical risk and economic policy uncertainty) on the connectedness as determined by the partial wavelet.

**Fig. 3:**
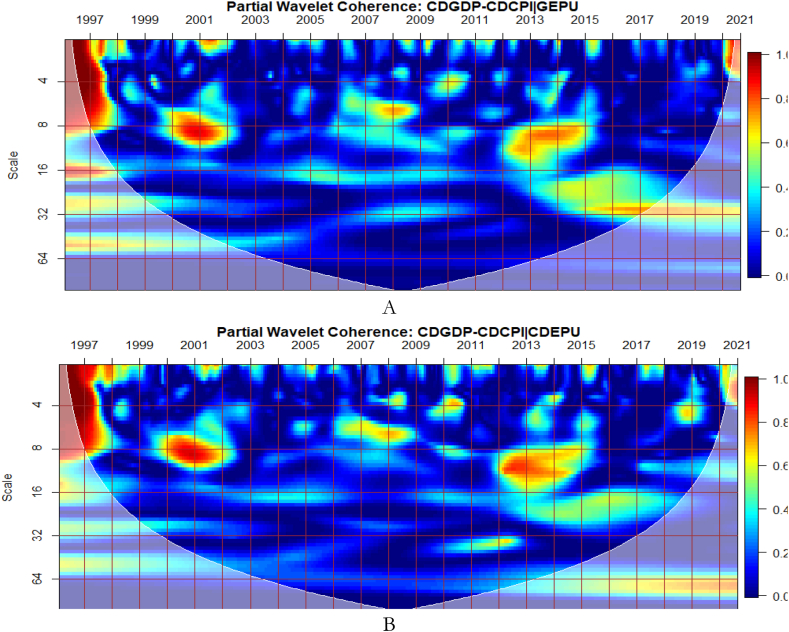

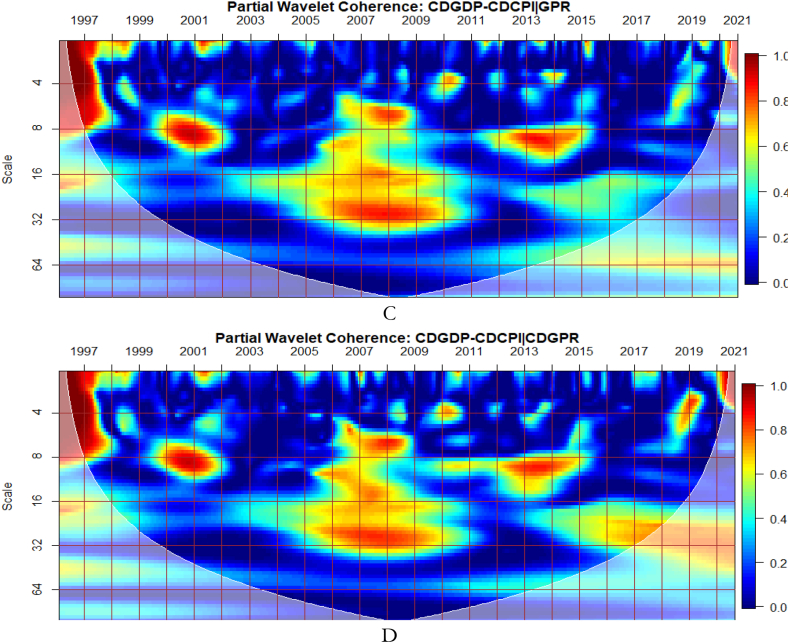
Partial impact of country level and global shocks on the co-movements between Canada's GDP and CPI. Panel A: CDGDP-CDCPI│GEPU; Panel B: CDGDP-CDCPI│CDEPU; Panel C: CDGDP-CDCPI│GPR; and Panel D: CDGDP-CDCPI│CDGPR. Note: The symbol │ denotes the partial/conditional impact of an uncertainty indicator.

The study presents the conditional impact of economic policy uncertainty and geopolitical risk in Fig. 3 (A and B) and Fig. 3 (C and D) respectively at both country level and global shocks on the co-movements between Canada's GDP and CPI. The EPU of Canada in Fig. 3(B) has the most substantial conditional impact on the co-movements between GDP and CPI. The strongest impact of EPU from Canada on the nexus is not surprising because policy uncertainty from the country disturbs the macroeconomy [54]. It is also known to have a ravaging influence on financial markets in the country [55,56]. This is closely followed by the conditional impact of GEPU in Fig. 3(A). Hence, the EPU of Canada is a major determinant of the GDP-CPI nexus relative to the GEPU and geopolitical risk. Conversely, the global geopolitical risk factor (GPR) is shown in Fig. 3 (C) to have a greater conditional impact on the nexus relative to the country-specific GPR in Fig. 3(D). This implies that wars, terrorist acts, and tensions-related risks among nations other than those merely accruing from Canada have a significant influence on GDP-CPI nexus.

**Fig. 4:**
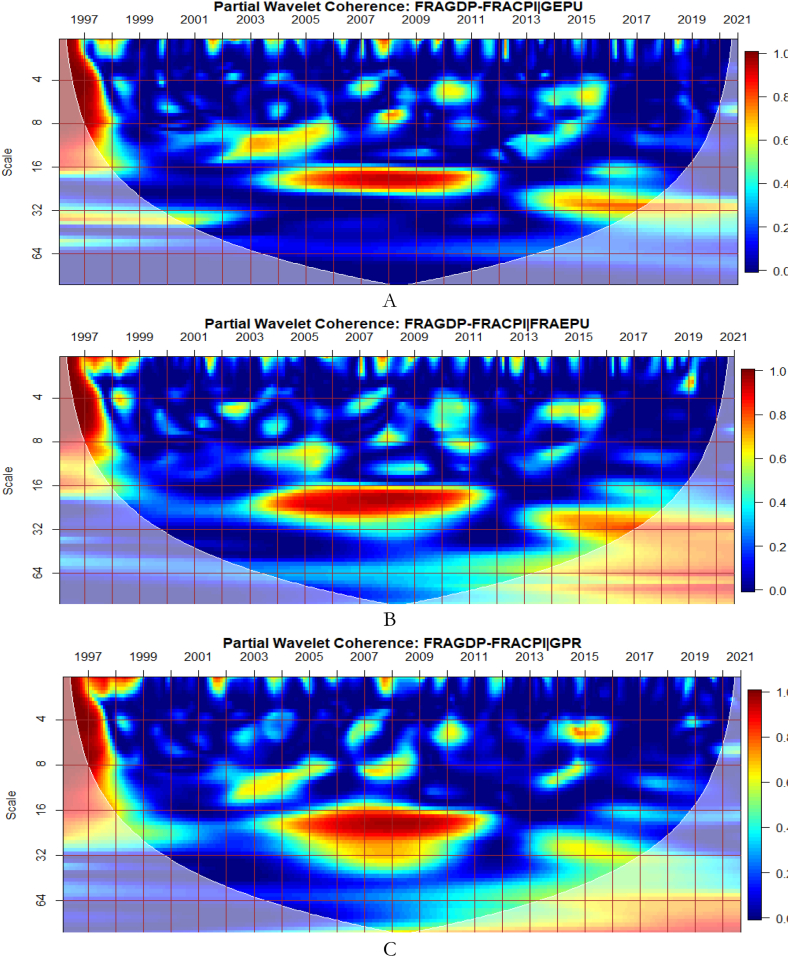

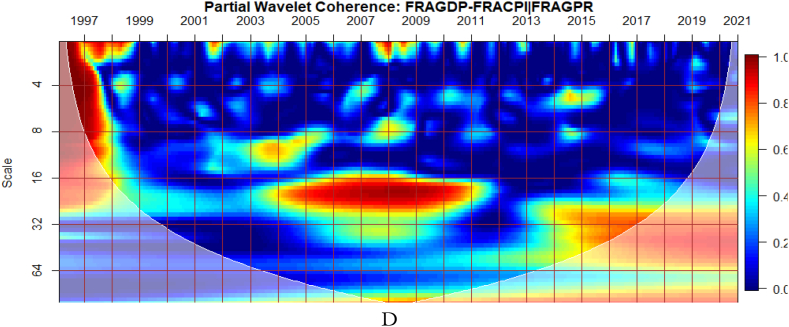
Partial impact of country level and global shocks on the co-movements between France's GDP and CPI. Panel A: FRAGDP-FRACPI│GEPU; Panel B: FRAGDP-FRACPI│FRAEPU; Panel C: FRAGDP-FRACPI│GPR; and Panel D: FRAGDP-FRACPI│FRAGPR. Note: The symbol │ denotes the partial/conditional impact of an uncertainty indicator.

From Fig. 4, we show the partial influence of EPU and GPR risk at both country level and global shocks on the co-movements between France's GDP and CPI. The EPU of France in Fig. 4(B) has the most significant conditional effect on the co-movements between GDP and CPI. However, from Fig. 4(A), the GEPU is noticeable to transmit significant conditional shocks between 2003 and 2012, exhibiting a channel through which GDP and CPI can be affected, especially in crisis-related period. Hence, the GEPU closely follows the risk transmission ability of the EPU of France. As revealed for Canada, the EPU of France in Fig. 4(B) and France's GPR in Fig. 4(D) is also a major determinant of the co-movements between GDP and CPI. Nonetheless, the conditional impact of global GPR in Fig. 4(C) is the strongest except for, between 2003 and 2011. The weaker conditional impact of GPR during this period is slightly attributed to the GFC. Hence, wars, terrorist acts, and tensions-related risks among nations act as a weaker channel to influence the GDP-CPI nexus during financial crisis.

**Fig. 5:**
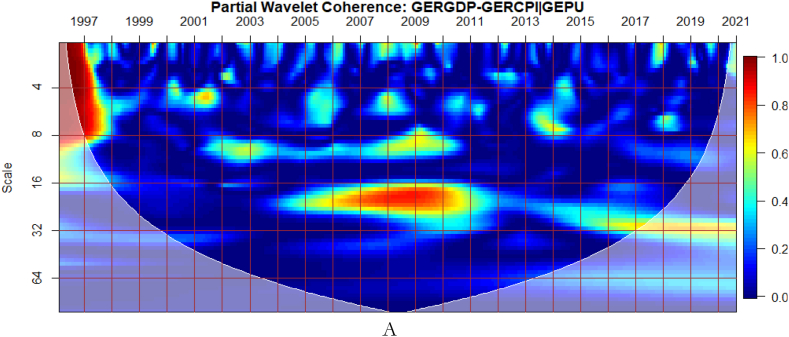

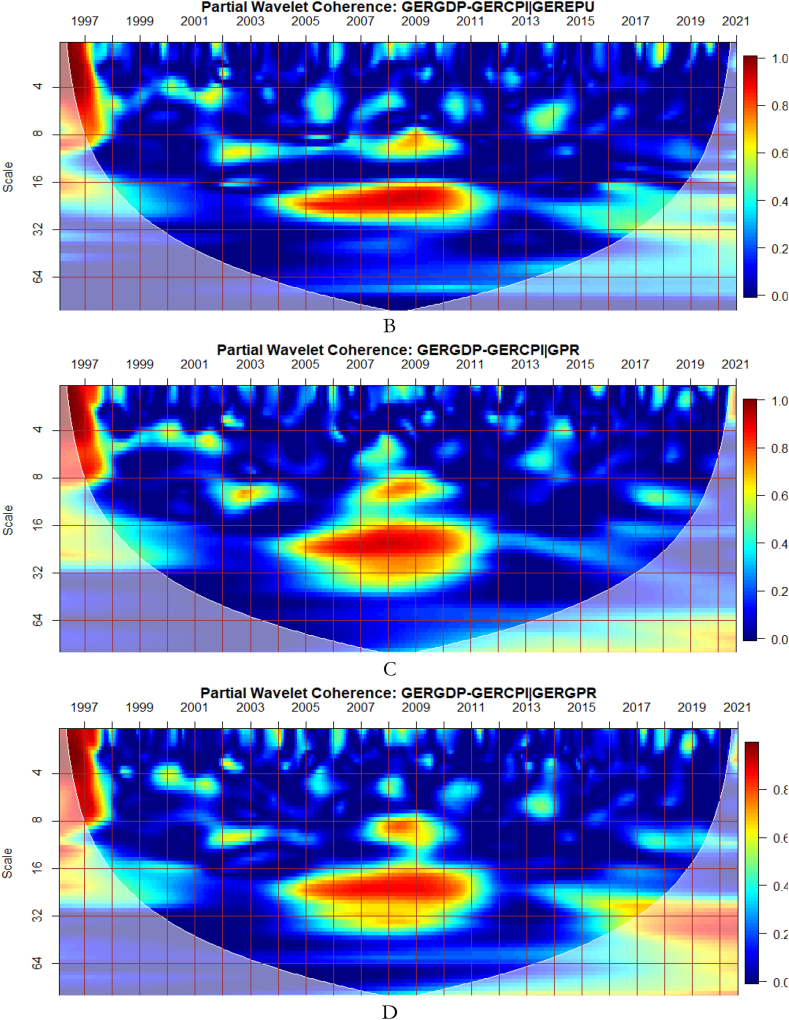
Partial impact of country level and global shocks on the co-movements between Germany's GDP and CPI. Panel A: GERGDP-GERCPI│GEPU; Panel B: GERGDP-GERCPI│GEREPU; Panel C: GERGDP-GERCPI│GPR; and Panel D: GERGDP-GERCPI│GERGPR.

It can be seen from Fig. 5(A) that GEPU is an important channel through which lead-lag relationship between GDP and CPI is mitigated, especially during the 2008 GFC. This is closely followed by the partial influence of EPU from Germany in Fig. 5(B). Additionally, wars, terrorist acts, and tensions-related risks among nations are significant transmitters of shocks relative to the ones directly associated with Germany as respectively shown in Fig. 5(C) and (D). This is consistent with the findings of Tunc, Kocoglu and Aslan [57] who indicated that GDP in Germany is susceptible to uncertainty shocks with a protracted influence during the 2008 Global Financial crisis across economic horizons.

**Fig. 6:**
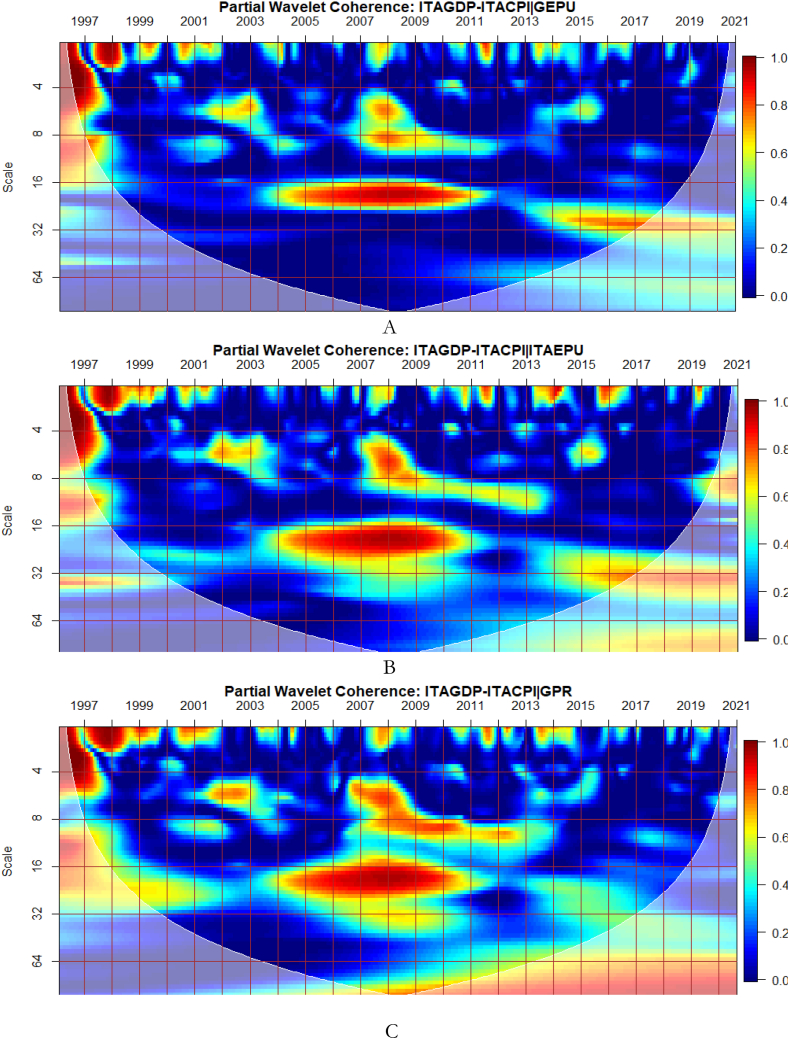

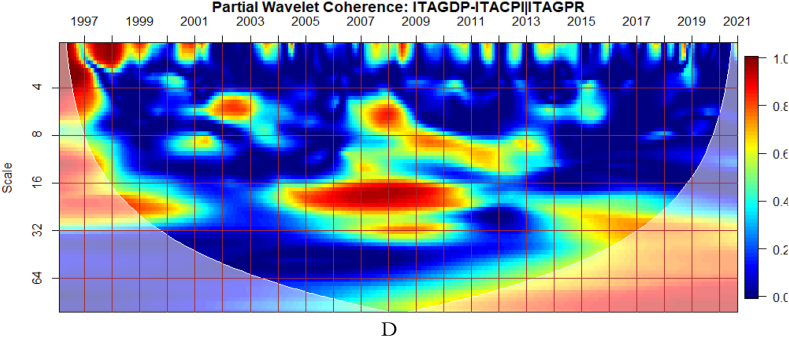
Partial impact of country level and global shocks on the co-movements between Italy's GDP and CPI. Panel A: ITAGDP-ITACPI│GEPU; Panel B: ITAGDP-ITACPI│ITAEPU; Panel C: ITAGDP-ITACPI│GPR; and Panel D: ITAGDP-ITACPI│ITAGPR. Note: The symbol │ denotes the partial/conditional impact of an uncertainty indicator.

From Fig. 6(A), GEPU is a strong channel that affects the co-movements between GDP and CPI, especially during the 2008 GFC. Accordingly, economic policy uncertainty has larger long-lived repercussions for Italy's embarkment to sustained economic growth, mitigate risks of inflation, and improve overall financial performance. This is closely followed by the conditional influence of EPU from Italy in Fig. 6(B). Policy uncertainty from Italy is found to have convoluting influence on a network of other sentiment indicators [58]. Moreover, in Fig. 6(C), the global GPR is a significant transmitter of shocks relative to the GPR from Italy presented in Fig. 6(D).

**Fig. 7:**
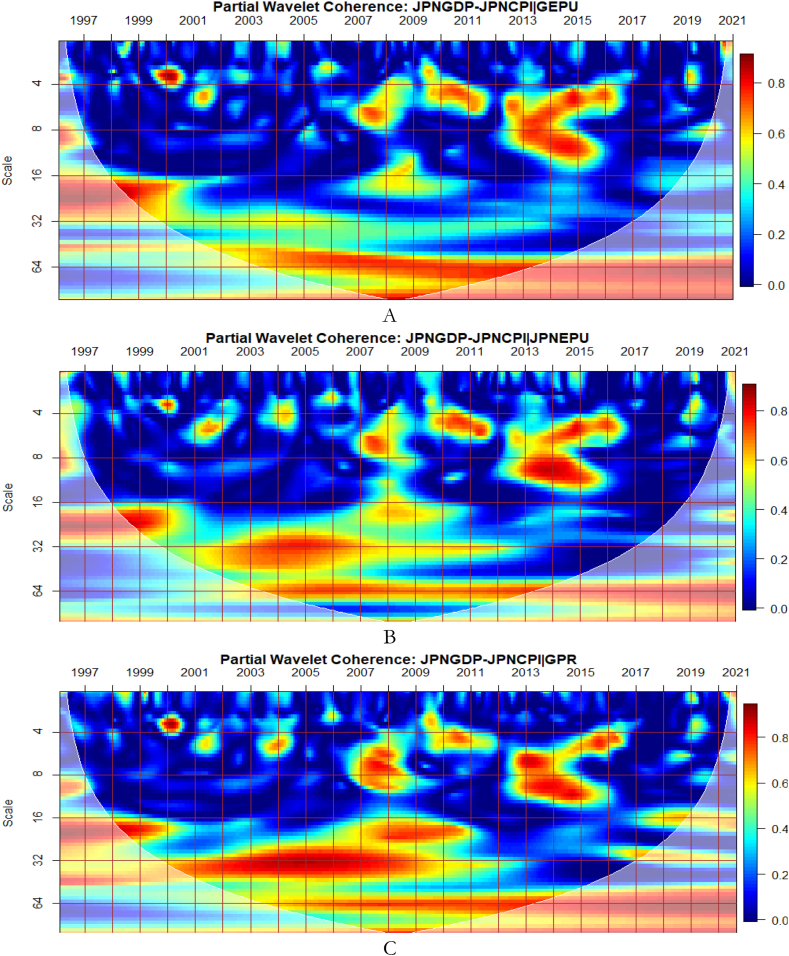

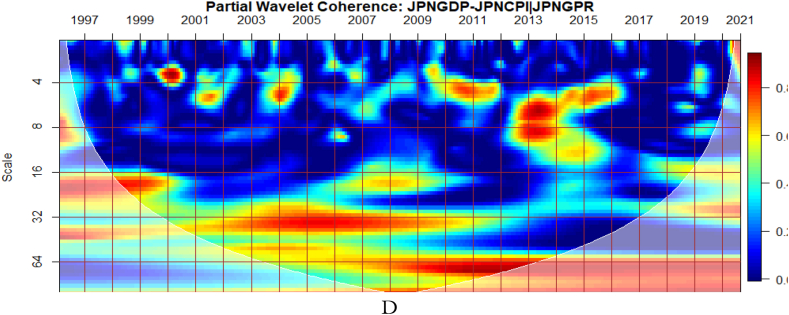
Partial impact of country level and global shocks on the co-movements between Japan's GDP and CPI Panel A: JPNGDP-JPNCPI│GEPU; Panel B: JPNGDP-JPNCPI│JPNEPU; Panel C: JPNGDP-JPNCPI│GPR; and Panel D: JPNGDP-JPNCPI│JPNGPR.

The co-movements between GDP and CPI are strong to withstand the conditional influence of EPU in Fig. 7 (A and B) and GPR emanating globally shown in Fig. 7(C) and internally as presented in Fig. 7(D). Accordingly, the deflation of the Japanese economy coupled with the significant impact of known economic events are important channels that contribute to the GDP-CPI nexus. However, during the 2008 Global Financial crisis, the conditional influence of the uncertainty shocks is severe on the GDP-CPI nexus. Accordingly, although Japan recorded deflation over decades to this period the crisis had a stronger protracted adverse influence on the real economy which triggered inflationary pressures. This outcome is particularly confirmed in the study of Adeosun et al. [59].

Comparatively, shocks from GEPU have the largest influence on the co-movements between GDP and CPI of Japan as can be found in Fig. 7(A). Moreso, policy uncertainty has a larger protracted challenge for Japan's embarkment to sustained economic growth, reduce the impact of deflation, and resuscitate national economic and financial performance. This is consistent with the study of Athari et al. [60] who revealed the heterogeneous and adaptive susceptibility of inflation in Japan to uncertainty. Hence, shocks to inflation are likely to hinder output and employment levels.

**Fig. 8:**
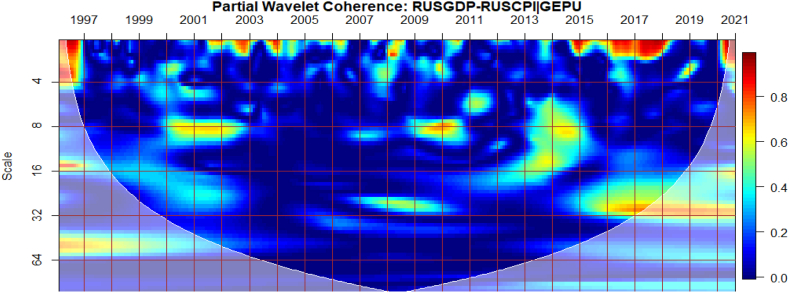

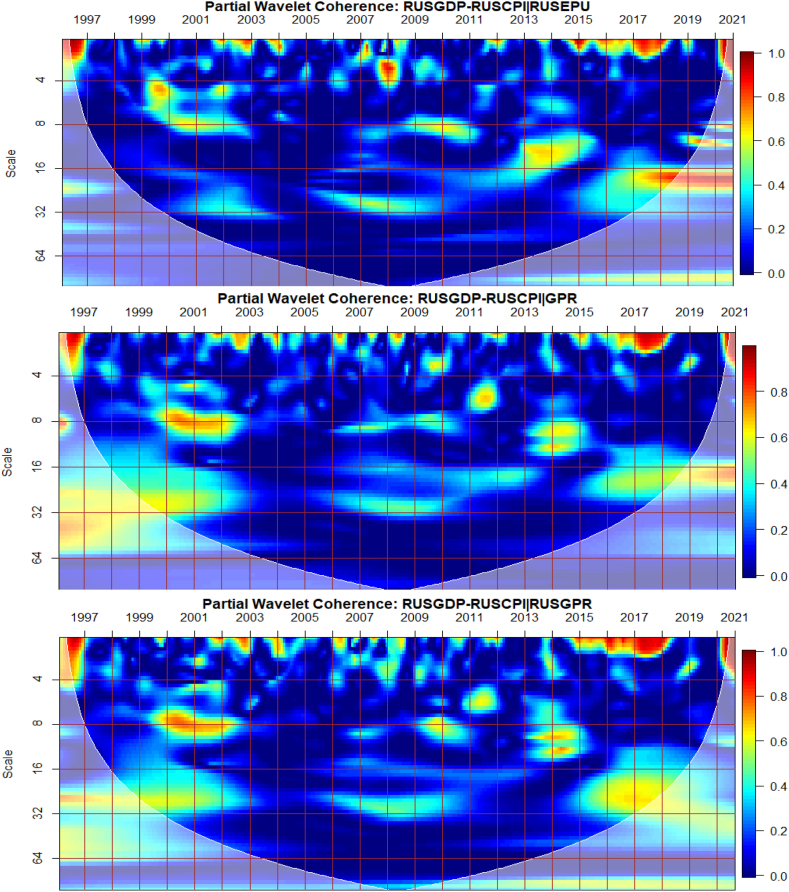
Partial impact of country level and global shocks on the co-movements between Russia's GDP and CPI, Panel A: RUSGDP-RUSCPI│GEPU; Panel B: RUSGDP-RUSCPI│RUSEPU; Panel C: RUSGDP-RUSCPI│GPR; and Panel D: RUSGDP-RUSCPI│RUSGPR. Note: The symbol │ denotes the partial/conditional impact of an uncertainty indicator.

It is noticeable from Fig. 8(C and D) that global and country-specific GPR factors have the most significant influence on the GDP-CPI nexus in Russia beyond 2002 relative to GEPU and EPU of Russia as respectively presented in Fig. 8(A and B). Hence, wars, terrorist acts, and tension-related risks are known to influence the co-movements between GDP and CPI of Russia. As found by Caldara et al. [30], fluctuations in GPR drive inflation whose impact, in turn, is crucial for output and growth, unlike the deflationary effect of plunging consumer expectations with stricter economic conditions which are in support of the outcome by Caldara et al. [30]. It, therefore, implies that country-specific GPR is inflationary with stronger implications for Russia's economic growth levels which has large military expenditure. Nonetheless, the existing nexus between GDP and CPI was found to be less integrated as mostly revealed by prior studies on the country's weak linked macroeconomic fundamentals [53,61]. However, due to Russia's major dependence on oil and the advanced stock market, significant integration of macroeconomic indicators mostly occurs with international crude oil prices and stock returns [38,42,49,62,63].

**Fig. 9:**
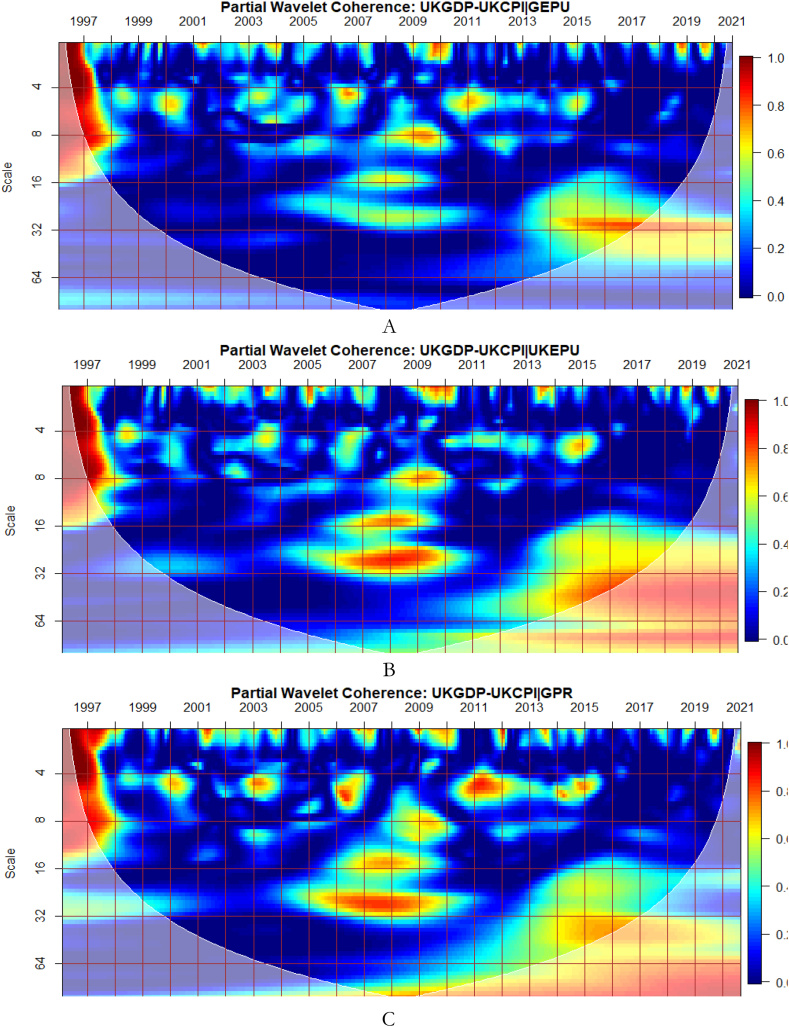

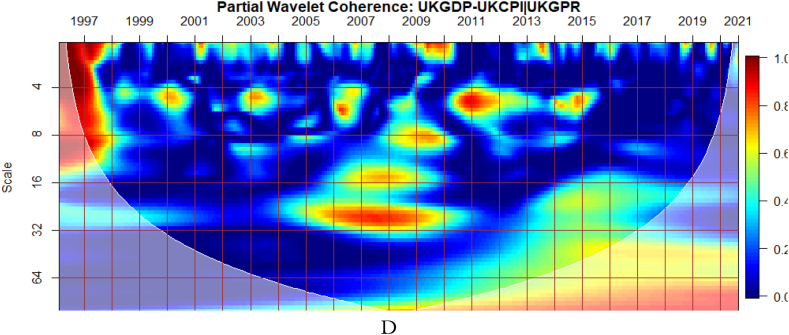
Partial impact of country level and global shocks on the co-movements between UK's GDP and CPI. Panel A: UKGDP-UKCPI│GEPU; Panel B: UKGDP-UKCPI│UKEPU; Panel C: UKGDP-UKCPI│GPR; and Panel D: UKDP-UKCPI│UKGPR. Note: The symbol │ denotes the partial/conditional impact of an uncertainty indicator.

As illustrated from Fig. 9(A), GEPU is an important channel through which co-movements between GDP and CPI are influenced, particularly during the 2008 GFC in UK. This is closely followed by the conditional effect of EPU from UK in Fig. 9(B). This is not startling because fluctuations in EPU have a stronger asymmetric effect on macroeconomic dynamics in the UK [55]. The results demonstrate that heighten uncertainty hinders the co-movements between GDP and CPI. This partly agrees with the outcome of Adedoyin and Zakari [64] that EPU matters most in economic growth of UK, but only in the short-term. However, in Fig. 9(D), GPR from UK has significant partial influence on the co-movements as compared to the global GPR in Fig. 9(C). This implies that GDP-CPI nexus of UK is greatly vulnerable to GEPU relative to country-specific EPU but responds largely to country-specific GPR than global GPR. Knowing that uncertainty indices have a large impact also on inflation dynamics in UK [59,65], the stronger conditional influence of these uncertainties on the GDP-CPI nexus is not surprising.

**Fig. 10:**
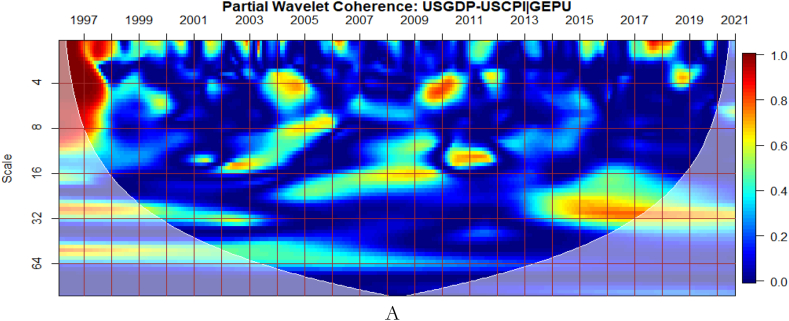

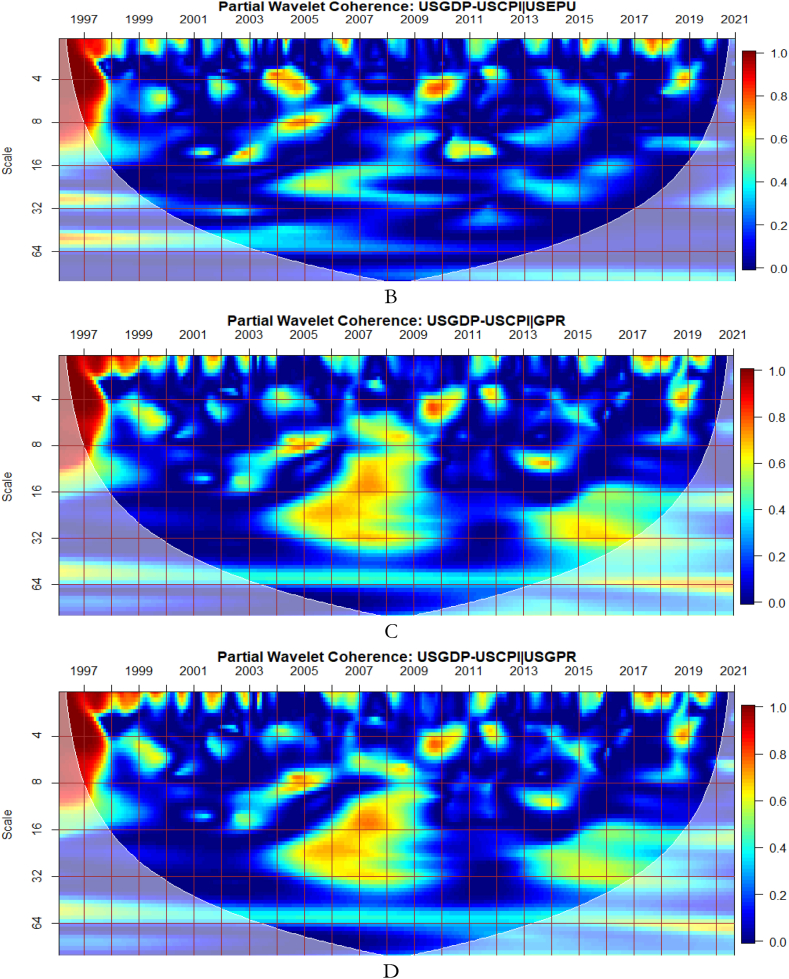
Partial impact of country level and global shocks on the co-movements between US's GDP and CPI. Panel A: USGDP-USCPI│GEPU; Panel B: USGDP-USCPI│USEPU; Panel C: USGDP-USCPI│GPR; and Panel D: USGDP-USCPI│USGPR.Note: The symbol │ denotes the partial/conditional impact of an uncertainty indicator.

From Fig. 10(B), the US EPU is significant enough to affect the co-movements between GDP and CPI across time and frequency relative to the GEPU in Fig. 10(A). This explains the fact that high economic policy uncertainty from the US is stronger to distort most economic activities [67–70. This is closely followed by the conditional effect of GEPU. Moreover, in Fig. 10(D), GPR from US has significant partial effect on the co-movements between GDP and CPI relative to the global GPR as presented in Fig. 10(C). It implies that country specific factors from the US have serious ramifications for economic indicators than what is offered under global shocks. In this manner, the discussion of macroeconomic fundamentals of US, it is relevant to consider US-based shocks emanating from her economy, policy, uncertainty, wars, terrorist acts, and tensions-related risks. This can also be seen from the contagious impact of shocks from the US to other economies around the globe [66,67].

### 4.4 Robustness

#### 4.4.1 DCC-GARCH connectedness

**Fig. 11:**
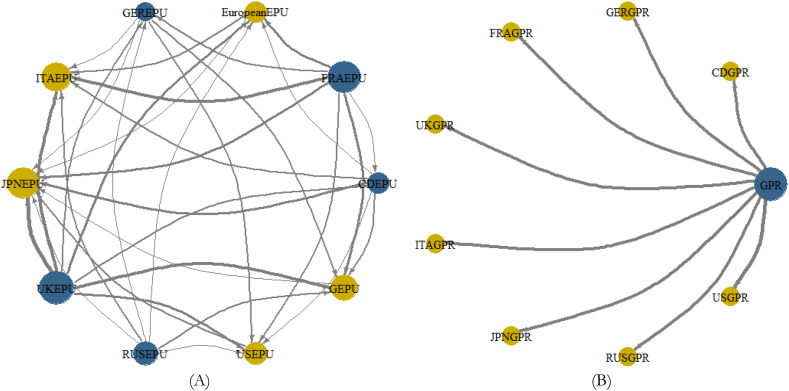
Net Pairwise Directional Connectedness for Panel A (connectedness among G8's EPU and GEPU) and Panel B (a network of G8's GPR and the global GPR connectedness). Note: The net directional connection between two variables is depicted by the arrows. While brown nodes describe shock net receivers, blue nodes illustrate shock net transmitters. The weighted average net total directional connectivity is represented by the nodes' sizes.

We seek to confirm the results of the wavelet approaches using the DCC-GARCH technique as developed by Gabauer [16]. Fig. 11 is a preliminary analysis to investigate potential net receivers and net transmitters of shocks from EPU and GPR. Therefore, Fig. 11 helps in the selection of dominant net receivers and net transmitters of shocks to be utilised for further empirical inquiry in the network of GDP and CPI.

From Fig. 11(A), dominant net receivers are the EPU of Italy and Japan whereas net transmitters include that of UK and France. The GEPU is added to capture lost dynamics of the connectivity representing 21 countries’ GDP-weighted average EPU indices. Since the global GPR drives the country-level GPR, we employ only the GPR which captures most of the dynamics as presented in Fig. 11(B). Consequently, the EPU of Italy, Japan, UK and France, GEPU and GPR are selected to further examine their connectedness in a network of GDP and CPI among the G8 countries. This is done not just to ensure a simplification of model but to screen out and deliver sound and pertinent economic policy direction and implication for the economies.

**Fig. 12:**
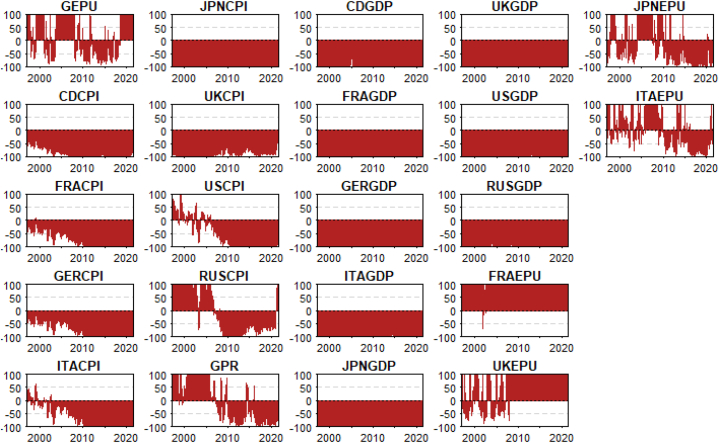
Net connectedness among GDP, CPI, GEPU, FRAEPU, ITAEPU JPNEPU, UKEPU and GPR

We proceed to examine the net connectedness among the selected macroeconomic variables for G8 economies by considering the difference between directional connectedness representing “To” and “From” as shown in Fig. 12. Hence, net connectedness permits a tendency for a shift between the roles of net transmitting and net receiving macroeconomic variables or both across time [68]. It must be noted that positive values signify net receivers of shocks whereas positive values suggest net transmitters. Macroeconomic variables that exhibit negative values throughout the period are classified as persistent net receivers whereas positive values, in this case, denote persistent net transmitters, and should be critically observed for economic decisions.

From Fig. 12, CPI and GDP for the G8 countries, but CPI of US, Russia and Italy are persistent net receivers of shocks. Prior to 2010, the average price change of US and Russia was net transmitters of shocks in a network of GDP, CPI, GEPU, FRAEPU, ITAEPU JPNEPU, UKEPU and GPR. In this manner, fluctuations in average price change from US and Russia may cause a contagion effect on other G8 countries. The net transmitting role of the CPI of US concurs the findings of Yang, Guo and Wang [69] that, unexpected fluctuations in the average price of the US have a large contagion effect on other G8 countries. This is not surprising because in terms of combating rising prices, the Federal Reserve Bank of the US rate hikes had a global impact. For instance, a rise in US interest rates is favourable to global investments which upsurge the dollar. This action then helps the US but weakens other economies rendering everything ensuing from actions like debt repayment and imports excessively expensive, and experiencing perience fluctuation in average prices of the US prior to 2010.

Moreover, as noted, since 1997, Russia's crisis and default shocked the global financial market with Russia being a significant borrower inducing economic and financial fragility of other economies. This led to a substantial rise in spreads on long-term corporate bonds in industrial countries. Accordingly, Russian economy which is highly dependent on oil, upon which other countries rely, weaker economic environment during this period would significantly cut the supply of oil by Russia. This makes oil prices expensive and contributes to increase in average price change, but initially captured in Russia's CPI leading to a contagion effect on other countries' economic fundamentals.

Despite the above, the increase in world economic and policy uncertainties, wars, terrorist acts, and tensions-related risks over the years has heightened the vulnerability of world economic activities. It can be seen that beyond, 2010, GDP and CPI of all G8 countries now exhibit net receiving roles with significant shocks transmitting from economic policy uncertainties at country and global levels. The EPU from France and UK beyond this point is a dominant transmitter of shocks which should be critically observed by the G8 economies. However, EPU from France is a persistent transmitter of shocks within the system which contradicts the findings of Bai et al. [66]. The net transmitting role of EPU and GPR arousing contagion effects concurs is consistent with the assertions made by Adeosun et al. [59] and Huang and Liu [55].

**Fig. 13:**
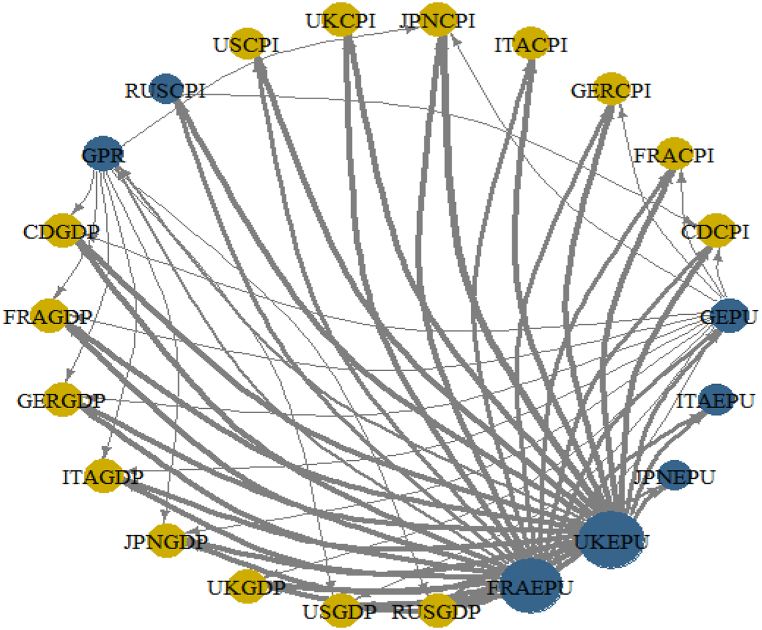
A network of Net Pairwise Directional Connectedness among GDP, CPI, GEPU, FRAEPU, ITAEPU JPNEPU, UKEPU and GPR. Note: Net directional connection between two variables is depicted by the arrows. While brown nodes describe shock net receivers, blue nodes illustrate shock net transmitters. The weighted average net total directional connectivity is represented by the nodes' sizes.

To reveal the extent to which external shocks could have convoluting influence on economic activities of the G8 countries, we present the network of Net Pairwise Directional Connectedness as shown in Fig. 13. In other words, Fig. 13 highlights the degree and direction of shocks received by GDP and CPI of the G8 countries from EPU and GPR. It further demonstrates shock transmission among the measures of external uncertainties within the same system. Moreover, we are able to decipher the static responses of the macroeconomic variables’ connectedness throughout the averaged sample period.

As shown in Fig. 13, GPR, GEPU, ITAEPU, JPEPU, UKEPU and FRAEPU are the net transmitters of uncertainty spillovers (blue coloured nodes) as partly found by Istrefi and Piloiu [34], the remaining variables are net receivers (brown coloured nodes). The influence of GPR on GDP corresponds to the assertion by Caldara and Iacoviello [28] and the outcome of Soybilgen et al. [31] that the geopolitical risk index captures events that are more exogenous to business and financial cycles, and could have serious ramification to incite economic instability.

Also, EPU of UK and France have the greatest connectedness (thick arrows). In particular, shocks transmitted from the EPU of UK to the EPU of France, Japan and Italy are in line with the findings of Kang and Yoon [70]. The dominance of the EPU of UK transmitting shocks to most macroeconomic factors partly agree with the outcome of Bai et al. [66]. All the G8's CPI, but Russia and GDP are net receivers of shocks for the sampled period in the system of the selected macroeconomic variables including external uncertainty shocks.

#### 4.4.2 Wavelet multiple cross-correlations

**Fig. 14:**
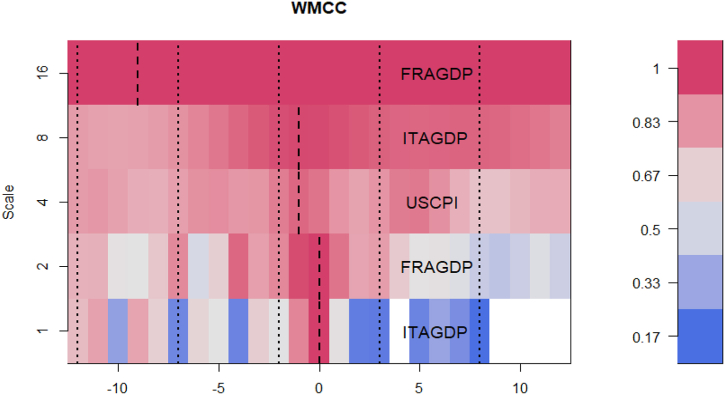
Wavelet multiple cross-correlations among GDP, CPI, GEPU, FRAEPU, ITAEPU JPNEPU, UKEPU and GPR

To respond to the difficulty of examining integration at frequency level, represent intrinsic times of short-, medium-, and long-terms, the wavelet multiple technique is utilised. This is shown in Fig. 14 and Table 2 as wavelet multiple cross-correlations (WMCC) among the selected macroeconomic variables. Hence, we are able to observe the degree of interdependencies among the variables and how they are connected across intrinsic time [[71], [72], [73], [74]]. Similar notions to those in the bi-wavelet scenario are represented by the scales on the y-axis of Fig. 14. However, the length of the series' lag is displayed on the x-axis. The positive and negative lags in this case are both twelve days. Positive localizations at the relevant scales denote lagging variables, while negative localizations denote leading variables. At the localization zero-lag, there is no lead or lag.

**Table 2:** WMCC coefficients among the network of exchange rate, energy commodities and volatilities.

Scale	Localisations	Time lags (days)	Leading/Lagging
1	0.999939375	0	ITAGDP
2	0.999667152	0	FRAGDP
3	0.929508592	−1	USCPI
4	0.985233067	−1	ITAGDP
5	0.99999837	−9	FRAGDP

The localization, which exhibits the highest values in the linear combination of all variables at the wavelet scales (at all lags), is shown by the dashed lines inside the dotted lines. The variable that is stated on a scale is the one that can either lead or lag the other variables. It implies that it has the highest value among all the variables at all the other scales when all the variables are linearly integrated at that scale. Because it demonstrates how closely related the variables are to one another and chooses the most important variable at a certain wavelet scale to act as a leading (first mover to respond to shocks) or trailing (latest variable to respond to shocks after the other variables) variable, the WMCC shows the impact on the economy. At a specific wavelet scale, the leading or trailing variable is influenced by the most significant variable, depending on how closely the other variables are related.

We find from Fig. 14 that GDP of Italy and France have the potential to lead or lag in the short-term. The dynamics changed in the medium-term where the CPI of US and GDP of Italy led. This implies that in the medium-term, USCPI and ITAGDP are the first variables to respond to outside shocks or act as first movers. Within the system of spillovers, shock transmission from USCPI and ITAGDP is eminent but only in the medium-term. The USCPI leading, in this case, is not surprising as unexpected variations in the average price of the US have a large contagion effect [69]. This contradicts the assertion by Adeosun et al. [59] where uncertainty indices (EPU and GPR) were inferred to lead inflation of the global players. However, in the long-term (scale 16), the GDP of France leads (at lag −9). Despite a recent decline in the GDP of France, it still has robust manufacturing, tourism, pharmaceuticals and significant agricultural resources with a high growth prospect. As indicated by the localisations, the system of selected macroeconomic variables is highly interdependent reaching as high as 99.9999% and as low as 99.9939%. The high connectedness among macroeconomic fundamentals of global players is consistent with the findings of Adeosun et al. [59] when the wavelet multiple coherence approach was used. We advocate that the variations in the dynamics interdependencies as depicted by different variables demonstrate heterogeneity.

## 5 Conclusion and policy recommendation

The study was investigated in three main folds. First, the co-movements between GDP and CPI of G8 countries were examined using the bi-wavelet approach. Second, the conditional influence of GPR and EPU was ascertained through the partial wavelet technique. Third, the degree and direction of connectedness among GDP and CPI of G8 in the midst of external shocks were determined through the DCC-GARCH Connectedness approach. We employed the wavelet cross-correlation to assess the magnitude of interdependence across intrinsic times of short-, medium-, and long-terms.

Findings from the study divulged that the nexus between GDP and CPI of most G8 countries are comparable. This corresponds to the economies’ capacity to foster harmony on global issues like economic growth, CPI and crisis management. Also, most of the significant co-movements between GDP and CPI of G8 countries span crises. Furthermore, geopolitical risk factors had a substantial conditional influence on the nexus between GDP and CPI of Russia. Own country economic policy uncertainty was the most significant shocks for countries like Canada, France and US whereas GEPU was a key channel through which lead-lag relationship between GDP and CPI is mitigated in Germany, Italy and UK. For Japan, there was no clear major determinant of external uncertainty shocks. Additionally, we found from the DCC-GARCH Connectedness approach, specifically, the Net Pairwise Directional Connectedness that in the network of selected macroeconomic variables, GDP and CPI of G8 economies except for CPI of Russia are net receivers of shocks.

Findings from this study imply that uncertainties have stronger protracted dynamic challenge for G8 countries' embarkment to a sustained economic growth, reduce the adverse impact of inflation and deflation, and restore national and regional economic performance. Firms postpone their investment and spending plans as a result of rising uncertainty [59]. The real-options effects have a negative effect on prices because firms eventually reduce production in response to weak demand, which pushes down inflation, and raises the unemployment rate [75]. In the face of shocks to the level of uncertainty, the labor markets may have an impact on employment and output. As a result, the heat of particular uncertainties, as revealed for each global player, has serious effects on economic growth dimensions and eventually causes economic growth to slow down. While firms may decide it is best to raise prices in response to contractionary uncertainty in order to avoid the risk of being forced to offer lower prices, shocks from EPU and GPR [59] may be transmitted to inflation through careful adjustment of national policies. However, high inflation also creates inefficiencies, dampens society's welfare, and ignites tensions related to geopolitical and policy uncertainty, which then lead to economic growth.

It can be concluded that GDP and CPI of G8 economies amid external uncertainty shocks are mostly connected exhibiting time-varying and frequency-dependent dynamics. The co-movements are degree of integration are found to be heterogeneous and adaptive. The country-specific GPR is more pronounced in the connectedness of economic growth and inflation of nations with induced military spending.

It is recommended to establish regional policies as a way of mitigation over an extended period to address the negative impact of economic policy uncertainties coming from France and the UK. These policies can be developed to the unique difficulties and dynamics of the impacted areas, offering focused solutions to reduce the detrimental spillover effects and encourage stability. Moreover, drawing conclusions from numerous economic indicators is essential to build, reengineer, and put into practice effective policies. The incidence of GPR, average price changes in Russia, GEPU, as well as economic policy uncertainty in France, Italy, Japan, and the UK, can all be examined in detail to gain important insights. Policymakers may better grasp the economic environment by adopting these insights, which will help them develop and create plans of action. Additionally, it is pertinent for the G8 countries’ monetary authorities to embark more deliberate policy coordination. This coordination could engage a shared policy objective centred around a long-term commitment to average price stability. By aligning their policies and objectives, these authorities can properly address global inflation and ease the influence of external uncertainty shocks and systemic crises for a sustainable economic growth.

The study was limited to G8 nations. Further studies may be conducted in other economic bodies such as G20 or regional blocs such as Southern Africa Development Community (SADC) to respond to global issues on the subject. Also, other internal risk factors which could have a protracted effect on the nexus were not considered. Accordingly, the roles of exchange rate and interest rate can be factored to contribute to the understanding of the macroeconomic modelling. Considering the rapid fluctuations in financial time series leading to their nonlinearities, analyses can also be performed using robust decomposition techniques coupled with the quantification of the amount of information that flows through the macroeconomic variables [[76], [77], [78], [79], [80], [81], [82]]. The sample period is limited to August 2021 due to limited data availability for some variables for the G8 nations but enough for the subject matter to be investigated. Hence, as a suggestion for future studies, an extended sample can be used to inculcate the systemic impact of the Russian-Ukrainian war for enhanced policy and practical decisions.

Emmanuel Asafo-Adjei: Thobekile Qabhobho: Anokye M. Adam: Conceived and designed the experiments; Performed the experiments; Analyzed and interpreted the data; Contributed reagents, materials, analysis tools or data; Wrote the paper. </p>

## Data availability statement

Data will be made available on request.

## Funding

No funding was received.

## Declaration of competing interest

The authors declare that they have no conflict of interest.

## References

[1] Vinayagathasan T. (2013). Inflation and economic growth: a dynamic panel threshold analysis for Asian economies. J. Asian Econ..

[2] Chimobi O.P. (2010). Inflation and economic growth in Nigeria. J. Sustain. Dev..

[3] Gokal V., Hanif S. (2004).

[4] Smal M.M. (1998). The cost of inflation. South African Reserve Bank Quarterly Bulletin.

[5] Sidrauski M. (1967). Inflation and economic growth. J. Polit. Econ..

[6] Fischer S. (1993). The role of macroeconomic factors in growth. J. Monetary Econ..

[7] Mallik G., Chowdhury A. (2001). Inflation and economic growth: evidence from South Asian countries. Asia Pac. Dev. J..

[8] Švigir M., Miloš J. (2017). Relationship between inflation and economic growth; comparative experience of Italy and Austria. FIP-Financije i pravo.

[9] Ebenezer O., Ogujiuba K., Maredza A. (2022). Exchange rate volatility, inflation and economic growth in developing countries: panel data approach for SADC. Economies.

[10] Baharumshah A.Z., Slesman L.Y., Wohar M.E. (2016). Inflation, inflation uncertainty, and economic growth in emerging and developing countries: panel data evidence. Econ. Syst..

[11] Leshoro T.L.L. (2012). Estimating the inflation threshold for South Africa. J. Stud. Econ. Econom..

[12] Phiri A. (2018). Nonlinear impact of inflation on economic growth in South Africa: a smooth transition regression analysis. Int. J. Sustain. Econ..

[13] Ramazan E., Osman T., Fatih C. (2020). The relationship between inflation and economic growth: experiences of some inflation targeting countries. Financial Studies.

[14] Umar Z., Bossman A., Choi S.Y., Teplova T. (2022). Does geopolitical risk matter for global asset returns? Evidence from quantile-on-quantile regression. Finance Res. Lett..

[15] Gourinchas (2022). Global economic growth slows amid gloomy and more uncertain outlook. Insights and analysis of economics and finance. JULY 26, 2022 [Blog].

[16] Gabauer D. (2020). Volatility impulse response analysis for DCC‐GARCH models: the role of Volatility Transmission Mechanisms. J. Forecast..

[17] Diebold F.X., Yılmaz K. (2014). On the network topology of variance decompositions: measuring the connectedness of financial firms. J. Econ..

[18] Junttila J., Vataja J. (2018). Economic policy uncertainty effects for forecasting future real economic activity. Econ. Syst..

[19] Liu Y., Zheng Y., Drakeford M. (2019). Reconstruction and dynamic dependence analysis of global economic policy uncertainty. Quantitative Finance and Economics.

[20] Rezaei N., Norouzi A. (2019). Investigating economic uncertainty and bank lending decisions. Quarterly Journal of Investment Knowledge.

[21] Haidarpour A., Pourshahabi F. (2012). Explaining the effects of economic uncertainty on macroeconomic variables (Case study: Iran). Quarterly Journal of Parliament and Strategy.

[22] Al-Thaqeb S.A., Algharabali B.G.H., Alabdulghafour K.T. (2020).

[23] Baker S.R., Bloom N., Davis S.J. (2016). Measuring economic policy uncertainty. Q. J. Econ..

[24] Fang L., Chen B., Yu H., Qian Y. (2018). The importance of global economic policy uncertainty in predicting gold futures market volatility: a GARCHMIDAS approach. J. Futures Mark..

[25] Flint C. (2016).

[26] Zhang Y., Hamori S. (2022). A connectedness analysis among BRICS's geopolitical risks and the US macroeconomy. Econ. Anal. Pol..

[27] Balcilar M., Bonato M., Demirer R., Gupta R. (2018). Geopolitical risks and stock market dynamics of the BRICS. Econ. Syst..

[28] Caldara D., Iacoviello M. (2022). Measuring geopolitical risk. Am. Econ. Rev..

[29] Alesina A., Perotti R. (1996). Income distribution, political instability, and Investment. Eur. Econ. Rev..

[30] Caldara D., Conlisk S., Iacoviello M., Penn M. (2023). Do geopolitical risks raise or lower inflation?. Tech. rep., Federal Reserve Board.

[31] Soybilgen B., Kaya H., Dedeoglu D. (2019). Evaluating the effect of geopolitical risks on the growth rates of emerging countries. Econ. Bull..

[32] Barros F., Gomes F.A.R., Soave G.P. (2022). Geopolitical risk shocks and the Brazilian economy. Appl. Econ. Lett..

[33] Wen J., Khalid S., Mahmood H., Yang X. (2022). Economic policy uncertainty and growth nexus in Pakistan: a new evidence using NARDL model. Econ. Change Restruct..

[34] Istrefi K., Piloiu A. (2016).

[35] Nyawo S.T., van Wyk R.B. (2018). The impact of policy uncertainty on macro-economy of developed and developing countries. J Eco Behav Stu.

[36] Farahani M.H., Ghabel S.N., Mohammadpour R. (2021). The effect of inflation threshold on financial development and economic growth: a case study of D-8 countries. Iran. Econ. Rev..

[37] Torrence C., Compo G.P. (1998). A practical guide to wavelet analysis. Bull. Am. Meteorol. Soc..

[38] Agyei S.K., Owusu Junior P., Bossman A., Asafo-Adjei E., Asiamah O., Adam A.M. (2022). Spillovers and contagion between BRIC and G7 markets: new evidence from time-frequency analysis. PLoS One.

[39] Friedman M. (1977). Nobel lecture: inflation and unemployment. J. Polit. Econ..

[40] Shin I., Kang K.H. (2021). Has international CPI inflation co-movement strengthened since the global financial crisis?. Macroecon. Dyn..

[41] Nkrumah-Boadu B., Owusu Junior P., Adam A.M., Asafo-Adjei E. (2022). Safe haven, hedge and diversification for African stocks: cryptocurrencies versus gold in time-frequency perspective. Cogent Economics & Finance.

[42] Amoako G.K., Asafo-Adjei E., Mintah Oware K., M Adam A. (2022). Do volatilities matter in the interconnectedness between world energy commodities and stock markets of BRICS?. Discrete Dynam Nat. Soc..

[43] Anghel M.G., Lilea F.P.C., Mirea M. (2017). Analysis of the interdependence between GDP and inflation. Romanian Statistical Review Supplement.

[44] Jing Z., Elhorst J.P., Jacobs J.P., de Haan J. (2018). The propagation of financial turbulence: interdependence, spillovers, and direct and indirect effects. Empir. Econ..

[45] Naghdi Y., Kaghazian S., Kakoei N. (2013). Effective comparison of global financial crisis (2007) on inflation of OPEC countries and selected countries of G8. Rom. Econ. J..

[46] Asafo-Adjei E., Frimpong S., Owusu Junior P., Adam A.M., Boateng E., Abosompim R.O. (2022). Multi-frequency information flows between global commodities and uncertainties: evidence from COVID-19 pandemic. Complexity.

[47] Kajitani Y., Chang S.E., Tatano H. (2013). Economic impacts of the 2011 Tohoku-Oki earthquake and tsunami. Earthq. Spectra.

[48] Taghizadeh-Hesary F., Yoshino N., Rasoulinezhad E. (2017). Impact of the Fukushima nuclear disaster on the oil-consuming sectors of Japan. J Comp Asian Dev.

[49] Asafo-Adjei E., Boateng E., Isshaq Z., Idun A.A.A., Owusu Junior P., Adam A.M. (2021). Financial sector and economic growth amid external uncertainty shocks: insights into emerging economies. PLoS One.

[50] Erdoğan S., Yildirim D.C., Gedikli A. (2020). Dynamics and determinants of inflation during the COVID-19 pandemic period in European countries: a spatial panel data analysis. Duzce MedJ.

[51] Hamidu Z., Oppong P.B., Asafo-Adjei E., Adam A.M. (2022). On the agricultural commodities supply chain resilience to disruption: insights from financial analysis. Math. Probl Eng..

[52] Qureshi F. (2022). COVID-19 pandemic, economic indicators and sectoral returns: evidence from US and China. Economic Research-Ekonomska Istraživanja.

[53] Bayramov V., Rustamli N., Abbas G. (2020). Collateral damage: the Western sanctions on Russia and the evaluation of implications for Russia's post-communist neighbourhood. Int Eco.

[54] Khan K., Su C.W. (2022). Does policy uncertainty threaten renewable energy? Evidence from G7 countries. Environ. Sci. Pollut. Control Ser..

[55] Huang W.Q., Liu P. (2022). Asymmetric effects of economic policy uncertainty on stock returns under different market conditions: evidence from G7 stock markets. Appl. Econ. Lett..

[56] Ma Y., Wang Z., He F. (2022). How do economic policy uncertainties affect stock market volatility? Evidence from G7 countries. Int. J. Finance Econ..

[57] Tunc A., Kocoglu M., Aslan A. (2022). Time-varying characteristics of the simultaneous interactions between economic uncertainty, international oil prices and GDP: a novel approach for Germany. Resour. Pol..

[58] Arreola Hernandez J., Kang S.H., Jiang Z., Yoon S.M. (2022). Spillover network among economic sentiment and economic policy uncertainty in Europe. Systems.

[59] Adeosun O.A., Tabash M.I., Vo X.V., Anagreh S. (2022). Uncertainty measures and inflation dynamics in selected global players: a wavelet approach. Qual. Quantity.

[60] Athari S.A., Kirikkaleli D., Yousaf I., Ali S. (2022). Time and frequency co‐movement between economic policy uncertainty and inflation: evidence from Japan. J. Publ. Aff..

[61] Balcilar M., Ike G., Gupta R. (2022). The role of economic policy uncertainty in predicting output growth in emerging markets: a mixed-frequency granger causality approach. Emerg. Mark. Finance Trade.

[62] Karim M.M., Chowdhury M.A.F., Masih M. (2022). Re-examining oil and BRICS’stock markets: new evidence from wavelet and MGARCH-DCC. Macroeconomics and Finance in Emerging Market Economies.

[63] Yuan D., Li S., Li R., Zhang F. (2022). Economic policy uncertainty, oil and stock markets in BRIC: evidence from quantiles analysis. Energy Econ..

[64] Adedoyin F.F., Zakari A. (2020). Energy consumption, economic expansion, and CO2 emission in the UK: the role of economic policy uncertainty. Sci. Total Environ..

[65] Choudhry T. (2023). Economic policy uncertainty and the UK demand for money: evidence from the inter-war period. J. Econ. Stud..

[66] Bai L., Zhang X., Liu Y., Wang Q. (2019). Economic risk contagion among major economies: new evidence from EPU spillover analysis in time and frequency domains. Phys. Stat. Mech. Appl..

[67] Zorgati I., Lakhal F., Zaabi E. (2019). Financial contagion in the subprime crisis context: a copula approach. N. Am. J. Econ. Finance.

[68] Idun A.A.A., Asafo-Adjei E., Adam A.M., Isshaq Z. (2022). Dynamic connectedness between indicators of the Ghana stock exchange returns and macroeconomic fundamentals. Risks.

[69] Yang J., Guo H., Wang Z. (2006). International transmission of inflation among G-7 countries: a data-determined VAR analysis. J. Bank. Finance.

[70] Kang S.H., Yoon S.M. (2019). Dynamic connectedness network in economic policy uncertainties. Appl. Econ. Lett..

[71] Armah M., Amewu G., Bossman A. (2022). Time-frequency analysis of financial stress and global commodities prices: insights from wavelet-based approaches. Cogent Economics & Finance.

[72] Asafo-Adjei E., Adam A.M., Idun A.A.A., Ametepi P.Y. (2022). Dynamic interdependence of systematic risks in emerging markets economies: a recursive-based frequency-domain approach. Discrete Dynam Nat. Soc..

[73] Boateng E., Asafo-Adjei E., Addison A., Quaicoe S., Yusuf M.A., Adam A.M. (2022). Interconnectedness among commodities, the real sector of Ghana and external shocks. Resour. Pol..

[74] Bossman A., Adam A.M., Junior P.O., Agyei S.K. (2022). Assessing interdependence and contagion effect on the bond yield and stock returns nexus in sub-saharan Africa: evidence from wavelet analysis. Scientific African.

[75] Phillips A.W. (1958). The relation between unemployment and the rate of change of money wage rates in the United Kingdom, 1861-1957. economica.

[76] Asafo-Adjei E., Adam A.M., Owusu Junior P., Arthur C.L., Seidu B.A. (2023). Multi-frequency information transmission among constituents and global equity returns: a sustainable and conventional way of investing. Eur. J. Manag. Bus. Econ..

[77] Armah M., Bossman A., Amewu G. (2023). Information flow between global financial market stress and African equity markets: an EEMD-based transfer entropy analysis. Heliyon.

[78] Boateng E., Asafo-Adjei E., Gatsi J.G., Gherghina Ş.C., Simionescu L.N. (2022). Multifrequency-based non-linear approach to analyzing implied volatility transmission across global financial markets. Oeconomia Copernicana.

[79] Umar Z., Bossman A., Choi S.Y., Vo X.V. (2023). Information flow dynamics between geopolitical risk and major asset returns. PLoS One.

[80] Asafo-Adjei E., Adam A.M., Arthur C.L., Seidu B.A., Gyasi R.M. (2022). Similarities among equities returns in multi-frequencies: insights from sustainable responsible investing. J sustain finance & investment.

[81] Qabhobho T., Asafo-Adjei E., Junior P.O., Adam A.M. (2022). Quantifying information transfer between commodities and implied volatilities in the energy markets: a multi-frequency approach. Int. J. Energy Econ. Pol..

[82] Boateng E., Owusu Junior P., Adam A.M., Abeka M., Qabhobho T., Asafo-Adjei E. (2022). Quantifying information flows among developed and emerging equity markets. Math. Probl Eng..

